# HnRNPA2 is a novel histone acetyltransferase that mediates mitochondrial stress-induced nuclear gene expression

**DOI:** 10.1038/celldisc.2016.45

**Published:** 2016-12-06

**Authors:** Manti Guha, Satish Srinivasan, Kip Guja, Edison Mejia, Miguel Garcia-Diaz, F Brad Johnson, Gordon Ruthel, Brett A Kaufman, Eric F Rappaport, M Rebecca Glineburg, Ji-Kang Fang, Andres Klein Szanto, Hiroshi Nakagawa, Jeelan Basha, Tapas Kundu, Narayan G Avadhani

**Affiliations:** 1 Department of Biomedical Sciences & Mari Lowe Center for Comparative Oncology, School of Veterinary Medicine, University of Pennsylvania, Philadelphia, PA, USA; 2 Department of Pharmacological Sciences, Stony Brook University, Stony Brook, NY, USA; 3 Department of Pathology and Laboratory Medicine, Perelman School of Medicine, University of Pennsylvania, Philadelphia, PA, USA; 4 Penn Vet Imaging Core, School of Veterinary Medicine, University of Pennsylvania, Philadelphia, PA, USA; 5 Vascular Medicine Institute, University of Pittsburg, Pittsburgh, PA, USA; 6 Nucleic Acid/Protein Core Facility, Children’s Hospital of Philadelphia Research Institute, Philadelphia, PA, USA; 7 Histopathology Facility, Fox Chase Cancer Center, Temple University, Philadelphia, PA, USA; 8 Department of Gastroenterology, Perelman School of Medicine, University of Pennsylvania, Philadelphia, PA, USA; 9 Transcription and Disease Laboratory, Molecular Biology and Genetics Unit, Jawaharlal Nehru Centre for Advanced Scientific Research, Jakkur, Bangalore, India

**Keywords:** acetyl-CoA binding domain, histone acetylation, hnRNPA2, Mitochondrial retrograde signaling (MtRS), mtDNA depletion, telomerase activation, transcriptional coactivator

## Abstract

Reduced mitochondrial DNA copy number, mitochondrial DNA mutations or disruption of electron transfer chain complexes induce mitochondria-to-nucleus retrograde signaling, which induces global change in nuclear gene expression ultimately contributing to various human pathologies including cancer. Recent studies suggest that these mitochondrial changes cause transcriptional reprogramming of nuclear genes although the mechanism of this cross talk remains unclear. Here, we provide evidence that mitochondria-to-nucleus retrograde signaling regulates chromatin acetylation and alters nuclear gene expression through the heterogeneous ribonucleoprotein A2 (hnRNAP2). These processes are reversed when mitochondrial DNA content is restored to near normal cell levels. We show that the mitochondrial stress-induced transcription coactivator hnRNAP2 acetylates Lys 8 of H4 through an intrinsic histone lysine acetyltransferase (KAT) activity with Arg 48 and Arg 50 of hnRNAP2 being essential for acetyl-CoA binding and acetyltransferase activity. H4K8 acetylation at the mitochondrial stress-responsive promoters by hnRNAP2 is essential for transcriptional activation. We found that the previously described mitochondria-to-nucleus retrograde signaling-mediated transformation of C2C12 cells caused an increased expression of genes involved in various oncogenic processes, which is retarded in hnRNAP2 silenced or hnRNAP2 KAT mutant cells. Taken together, these data show that altered gene expression by mitochondria-to-nucleus retrograde signaling involves a novel hnRNAP2-dependent epigenetic mechanism that may have a role in cancer and other pathologies.

## Introduction

The mitochondrial genome and its function are highly sensitive to environmental toxins, drugs and food additives. In addition, reduction in mitochondrial DNA (mtDNA) copy number and associated changes in mitochondrial membrane potential (Δψm) and mitochondrial dysfunction are common in numerous pathophysiological conditions, such as aging, cancer, neurodegenerative diseases and cardiomyopathy [1]. Dysfunctional mitochondria initiate retrograde signaling that is propagated through multiple mechanisms, including the Ca^2+^/calmodulin activated calcineurin pathway, mitochondrial reactive oxygen species (ROS)-induced hypoxia inducible factor (HIF) pathway, mitochondrial unfolded protein response or an AMPK activation pathway [2–6]. Mitochondrial retrograde signaling (MtRS) has been reported in different pathological conditions, including myoclonic epilepsy with ragged-red fibers (MERRF), diabetes, aging, cancer and deafness [7–11]. Notably, MtRS is intimately connected to disruption of mitochondrial membrane potential at its point of initiation and a shift in cellular metabolism as part of survival strategy through altered nuclear gene expression that determines cell fate [12]. However, the molecular mechanism underlying these processes remains unclear.

Compelling evidence from several studies supports the involvement of MtRS in tumor progression and cancer metastasis [13–16]. We have reported that in response to reduced mtDNA copy number the retrograde signaling is initiated by increased cytosolic [Ca^2+^]_c_ and the activation of protein phosphatase, calcineurin (Cn). In addition, reducing the mtDNA copy number in skeletal myocyte C2C12 cells below a certain threshold level (hereafter termed partial mtDNA depletion or PmtDNA depletion) causes metabolic shift to glycolysis, resulting in the emergence of tumorigenic cells from otherwise non-tumor forming immortalized cells [2, 13, 15, 17, 18]. In PmtDNA-depleted C2C12 cells, the induced activation of *Akt1,* glucose transporter *(Glut4),* ryanodine receptor *(RyR1),* and cathepsin L promoters depends on the activation of nuclear transcription factors *NFκB, C/EBPδ, CREB* and *NFAT*. We previously showed that these transcription factors were brought together to form an enhanceosome complex at the target promoters by a stress-induced RNA binding protein, heterogeneous ribonucleoprotein A2 (hnRNPA2). HnRNPA2 itself is activated in response to mtDNA depletion and acts as a transcriptional coactivator to propagate the signaling [19–21]. Interestingly, several studies suggest that hnRNPA2 is important in tumor progression and proliferation [22, 23].

Histone acetylation is a reversible epigenetic modification for transcriptional regulation. Histone lysine acetyltransferases (KATs) are enzymes that can transfer an acetyl moiety from acetyl-CoA and acetylate ε-lysine on the histone side chains [24]. Cellular and environmental cues activate KATs to alter the dynamic state of chromatin and ultimately determine the transcriptional fate of genes [25]. As many transcriptional coactivators are KAT proteins or recruit other KATs during active gene transcription [26], we investigated its role in transcription activation of stress-target promoters.

In this study, we show that altered mitochondrial function and its associated stress signaling have a causal role in transcriptional and epigenetic reprogramming of nuclear gene expression. We also report that hnRNPA2 is a novel mitochondrial stress-activated KAT, which acetylates histones on the MtRS target gene promoters resulting in altered epigenetic status of the cells. In addition, our results show that mitochondrial dysfunction-induced stress is a novel cellular cue for telomerase activation in immortalized cells. In a broader context, our findings here point to an important role for mitochondria as determinants of cell fate by altering the expression of large sets of nuclear genes through epigenetic mechanisms.

## Results

### H4 is acetylated at MtRS target gene promoters

**Figure 1:**
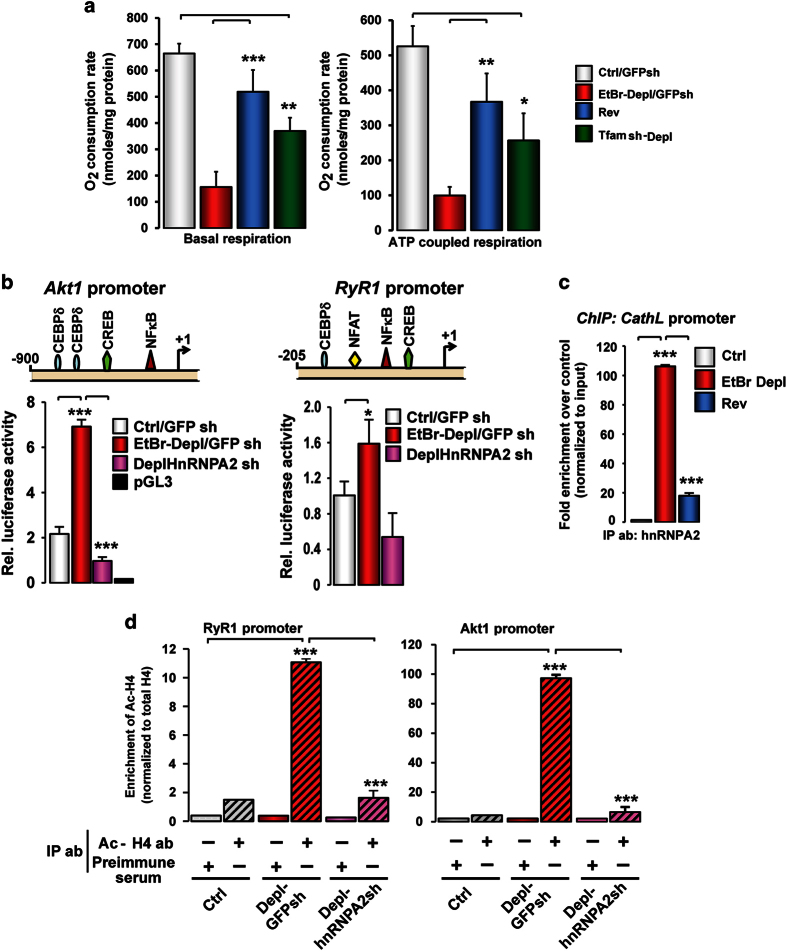
HnRNPA2-dependent histone H4 acetylation at stress-target gene promoters in mtDNA-depleted cells. (**a**) Effects of PmtDNA depletion on cellular respiration. (Left panel) Basal cellular respiration indicated as the oxygen consumption rate of parental, PmtDNA-depleted (EtBr-treated or *Tfam* shRNA) C2C12 cells measured on Seahorse XF24 analyzer using 50 000 cells per type. (Right panel) ATP-coupled and maximal respiration measured by sequential addition of oligomycin (2 μg ml^−1^), 3,5 dinitrophenol (DNP) (75 μm) and rotenone (1 μm), respectively. Data are represented as mean±s.d. (**b**) (Top panels) Cartoons of the mitochondrial stress-responsive *RyR1* and *Akt1* promoter regions depicting the putative mitochondrial stress-induced transcription factor binding sites mapped using the MatInspector algorithm. (Bottom panels) Promoter luciferase activities of stress-target genes, *Akt1* and *RyR1* after 48 h transfections in control (parental C2C12 cells), PmtDNA-depleted/mock-shRNA and PmtDNA-depl/hnRNPA2sh C2C12 cells. *pGL3* basic vector was used as negative control. Renilla luciferase activities were used for normalization of transfection efficiency. Data represent mean±s.d. (**c**) ChIP assay showing association of hnRNPA2 at cathepsin L promoter in control (parental C2C12 cells), PmtDNA-depleted and reverted C2C12 cells using hnRNPA2 antibody. (**d**) ChIP assay of *RyR1* and *Akt1* promoters from control, PmtDNA-depl, and PmtDNA-depl/hnRNPA2sh cells immunoprecipitated using an antibody to hyperacetylated histone H4. Data are represented as mean±s.d.

MtDNA content was reduced by either EtBR treatment or Tfam short hairpin RNA (shRNA) as described in ‘Materials and Methods’ section (Supplementary Figure S1A,B, D,E). Reduction in mtDNA copy number caused marked reduction in basal and ATP-coupled respiration, indicative of mitochondrial dysfunction in C2C12 cells (Figure 1a). As mentioned earlier, MtRS modulates the expression of a set of stress-target genes resulting in an acquired proliferative and oncogenic phenotype [13, 15, 19, 21]. Previously, we identified the minimal promoter regions of the MtRS target genes, m*RyR1* (−205 to +1) and m*Akt1* (−900 to +1), and showed that these regions contain *cis*-DNA elements required for mitochondrial stress response [19, 21]. Here, we show that these promoters were transcriptionally activated in PmtDNA-depl cells and this activation was diminished in cells stably expressing shRNA against hnRNPA2 (Figure 1b). Chromatin immunoprecipitation (ChIP) analysis shows that association of hnRNPA2 with the stress-target gene promoters cathepsin L, RyR1 and Akt1 was markedly increased in PmtDNA-depl cells, which was reduced by ~80% in reverted cells (Figure 1c; Supplementary Figure S3B).

As transcription activation and histone acetylation are interrelated, we investigated histone acetylation on the promoter regions of control, PmtDNA-depl cells and PmtDNA-depl cells stably expressing hnRNPA2shRNA (PmtDNA-depl/hnRNPA2sh). ChIP assays showed marked elevation of H4 acetylation (6-fold increase in *RyR1* and ~50-fold in *Akt1* promoter) in PmtDNA-depl cells compared with control cells (Figure 1d). Notably, this hyperacetylation was lost by shRNA-mediated knockdown of hnRNPA2, suggesting that H4 hyperacetylation of target gene promoters in response to MtRS may be mediated by hnRNPA2.

### Telomerase genes are targets of MtRS-activated hnRNPA2

**Figure 2:**
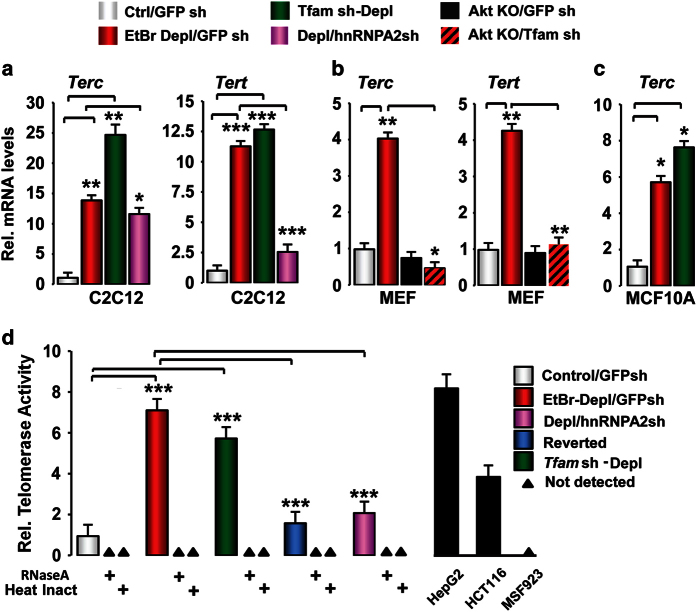
PmtDNA depletion induces telomerase activation by hnRNPA2. (**a**) Transcript levels of *Tert* and *Terc* in control, PmtDNA-depl and PmtDNA-depl/hnRNPA2sh C2C12 cells assessed by real time PCR. (**b**) *Terc* and *Tert* transcript levels in wild-type (WT) and protein kinase B/AKT kinase (AKT) knockout (KO) mouse embryonic fibroblast (MEF) parental and *Tfam* shRNA cells. (**c**) Transcript levels of *Terc* in parental and PmtDNA-depl MCF10A cells assessed by real time PCR. (**d**) Q-TRAP assay for telomerase activity was assessed in total cell extracts (1 μg protein) from control, PmtDNA-depleted (EtBr-treated or *Tfam* shRNA), PmtDNA-depl/hnRNPA2sh and reverted C2C12 cells. Cell extracts were treated either with RNAse or heat inactivated for negative controls. The telomerase expressing cancer cell lines HepG2 and HCT116 are used as positive controls and *Terc* null msf 923 cell lysate was used as a negative control. Data are represented as mean±s.d.

One hallmark of highly proliferative cancer cells is activation of telomerase to maintain the critical telomere length required to prevent cells from going into senescence. We previously demonstrated that hnRNPA2 acts as a transcriptional coactivator for several mitochondrial stress-target genes by associating with the enhanceosome complex at their promoters [19, 21]. Partial depletion of mtDNA in C2C12, MCF10A and MEF cells induced MtRS target genes (Supplementary Figure S1C) and proliferative phenotype [2, 13, 15, 19, 21]. This prompted us to consider the possibility that hnRNPA2 transcriptionally regulates telomerase by which mtDNA-depleted cells evade senescence and acquire oncogenic phenotype (Figure 2a). We observed marked increase in Telomerase RNA Component (*Terc)* and Telomerase reverse transcriptase (*Tert)* transcripts (10–20-fold) in PmtDNA-depl C2C12 cells, whereas this effect was diminished (50–75%) in PmtDNA-depl/hnRNPA2sh cells (Figure 2a). The increased transcription of *Terc* and *Tert* in PmtDNA-depl cells was also observed in MCF10A and MEFs (Figure 2b and c). Notably, *Tert* and *Terc* activation in PmtDNA-depl cells was Akt-dependent, further supporting the role of MtRS (Figure 2b).

Quantitative telomeric repeat amplification protocol (Q-TRAP) assay showed that PmtDNA-depl cell extracts exhibited ~6–7-fold higher telomerase activity, whereas in reverted cells the telomerase activity was similar to control cells, demonstrating a causal role of PmtDNA depletion in telomerase activation (Figure 2d). Skin fibroblasts from *Terc* null mice (msf 923) [27], used as negative controls showed no detectable activity (Figure 2d). Notably, PmtDNA-depl/hnRNPA2sh cells showed marked reduction in telomerase activity confirming that hnRNPA2 regulates telomerase gene expression in these immortalized cells with impaired mitochondrial function.

### HnRNPA2 is a histone lysine acetyltransferase (KAT) and Akt augments its function

**Figure 3:**
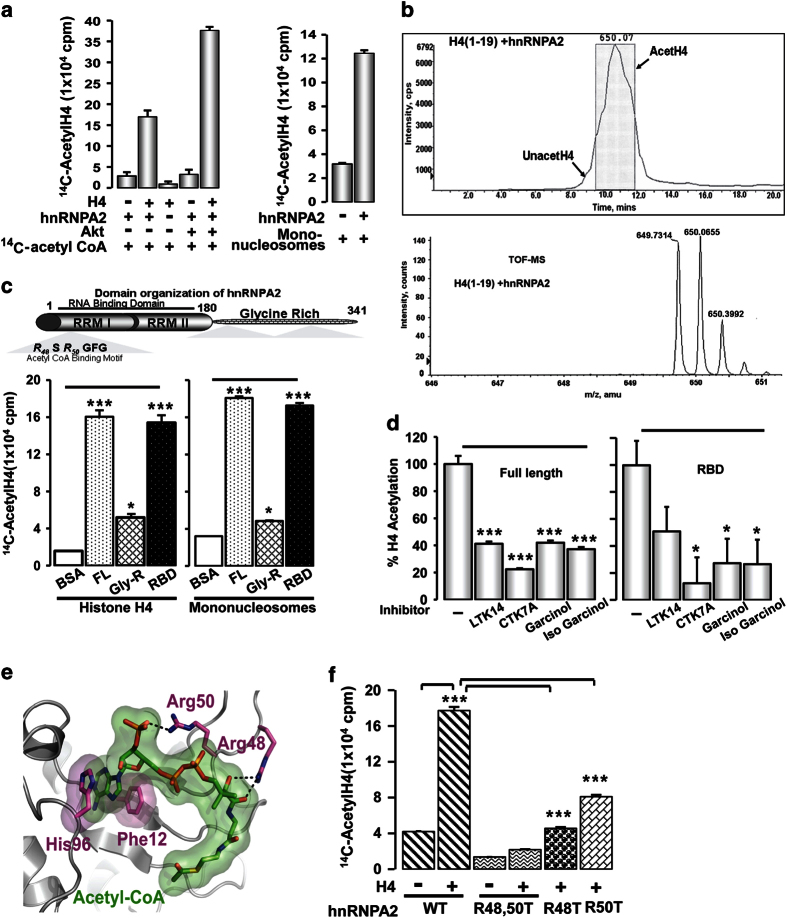
Characterization of KAT activity of hnRNPA2. (**a**) (Left panel) KAT activity of purified ×6 His-HnRNPA2 measured by a filter-binding assay using H4 peptide (residues 1–19) and ^14^C Acetyl-CoA as substrates (Supplementary Figure S2). Recombinant Akt kinase (Millipore, Cat # 14–276) was used to phosphorylate hnRNPA2 before the filter-binding assay as indicated (Supplementary Figure S2I). (Right panel) Filter-binding assay showing acetylation using mononucleosomes as substrate. (**b**) (Top) Liquid chromatography–mass spectrometry (LC-MS) profile of hnRNPA2-mediated acetylation status using histone H4 (synthetic H4 1–19 residues). (Bottom) Chromatogram showing the total ion counts of acetylated H4 (residues 1–19) peptide after reaction with hnRNPA2. The *X*-axis represents the liquid chromatography running time and the *Y*-axis represents the ion counts. (Bottom panel) The peak in the top panel was highlighted to generate the TOF-MS bottom panel. (**c**) (Top) Domain architecture of hnRNPA2. (Bottom) KAT activity of ×6 His-HnRNPA2 (full-length), RNA binding domain (RBD) (residues 1–180) and glycine-rich domain (residues 180–341) assessed by filter-binding assay using ^14^C acetyl-CoA and H4 peptide (residues 1–19) as substrates. (**d**) Inhibition of H4 acetylation by hnRNPA2 (full-length and RBD) using ^14^C acetyl-CoA in the presence of 100 μm synthetic KAT inhibitors (as indicated). (**e**) Proposed model for acetyl-CoA binding by hnRNPA2. A homology model of hnRNPA2 based on the hnRNPA1 structure (gray) with acetyl-CoA docked (green; see ‘Materials and Methods’ section) suggests that the adenosine base is sandwiched between two aromatic residues (H96 and F12; magenta), whereas additional interactions are established with Arg 48 and Arg 50 (magenta). (**f**) KAT activity of the purified full-length hnRNPA2 WT and R48T and R50T mutants using ^14^C Acetyl-CoA and H4 peptide (residues 1–19) as substrates. For LTK14, garcinol and isogarcinol DMSO alone was used in control assays run alongside.

Results in Figure 1d raised the possibility that hnRNPA2 either has intrinsic KAT activity or regulates other KATs *in vivo*. We therefore tested if purified hnRNPA2 can catalyze protein acetylation. As shown in Figure 3a, purified recombinant hnRNPA2 acetylated histone H4 as seen from the filter-binding assays using ^14^C-labeled acetyl-CoA. We further confirmed the KAT activity of hnRNPA2 by additional approaches such as filter-binding assays using fluorescent probes, ELISA (using anti-acetyl lysine antibody) using *in vitro* translated hnRNPA2 in a transcription-linked translation system as well as recombinant purified (<85% pure) proteins (Supplementary Figure S2A–C). Notably, hnRNPA2 acetylated mononucleosomes as assessed by a filter-binding assay (Figure 3a, right panel). Furthermore, the KAT activity of hnRNPA2 was protein concentration-dependent and was abrogated following either heat inactivation or repeated freeze-thawing (Supplementary Figure S2D and G) confirming its enzymatic nature.

HnRNPA2 binds to acetyl-CoA in a self-catalyzed reaction and is autoacetylated. In a filter-binding assay, we found that in the presence of excess ^3^H acetyl-CoA (50 μm), hnRNPA2 underwent autoacetylation (Supplementary Figure S2E). Extensive hnRNPA2 autoacetylation, which increased with increasing enzyme and acetyl-CoA concentrations, was evident from the highly immunoreactive bands of the acetyl lysine antibody (Supplementary Figure S2F). Similar to p300, MOF and Rtt109, autoacetylation of hnRNPA2 possibly augments its KAT activity by conformational changes following autoacetylation [29–33]. We previously reported that MtRS activation of hnRNPA2 involves Akt-mediated phosphorylation of hnRNPA2 at Thr98 and Ser219, which is essential for the former’s transcriptional coactivator function [21]. Therefore, we assessed the effect of Akt phosphorylation on the hnRNPA2 KAT activity. *In vitro* phosphorylation of hnRNPA2 by Akt enhanced its intrinsic KAT activity by ~2.5-fold over its non-phosphorylated state (Figure 3a). In addition, Akt phosphorylation site mutant hnRNPA2 proteins (T98A and S219A) showed ~40–50% reduced KAT activity (Supplementary Figure S2I) confirming that Akt-mediated phosphorylation is important for hnRNPA2 KAT activity. This is reminiscent of p300 where Akt-mediated phosphorylation is necessary for its KAT activity and transcriptional coactivator function [34].

The *in vitro* acetylation of H4 was further confirmed by LC/MS analysis of a synthetic H4 peptide (residues 1–19) (Figure 3b). The MS-TOF patterns presented in Figure 3b show that the H4 peptide was acetylated only in the presence of hnRNPA2 and the peptide alone did not show any detectable acetylation. Importantly, the KAT activity was unique to hnRNPA2, as hnRNPA1, a close relative of hnRNPA2 did not show this KAT activity (Supplementary Figure S2G). Notably, only hnRNPA2, but not hnRNPA1 was induced in PmtDNA-depleted cells (Supplementary Figure S2H).

To identify the regions of hnRNPA2 critical for its KAT function, we expressed a series of deletion constructs in E. coli and purified the recombinant His-tagged proteins (Coomassie Blue Staining for purified protein profile shown in Supplementary Figure S2C). The RBD of hnRNPA2 (residues 1–180) showed KAT activity comparable with the full-length protein *in vitro*, whereas the C-terminal glycine-rich domain (residues 180–341) showed no appreciable KAT activity with same molar amounts of protein (Figure 3c).

We tested the effects of an array of synthetic KAT inhibitors on hnRNPA2 KAT activity, including garcinol and garcinol derivatives (isogarcinol and LTK14) and hydrazinocurcumin CTK7A [35,36]. The KAT activity of hnRNPA2 was markedly inhibited by all four inhibitors (Figure 3d). A substantial inhibition of KAT activity by CTK7A suggests that hnRNPA2 possibly shares significant structural and/or catalytic center homologies with other KATs, particularly p300. The inhibition was dose dependent, and lower doses of 25 μm (data not shown) and 50 μm also showed inhibition (Supplementary Figure S2J).

### Histone H4 acetylation is mediated by RBD of hnRNPA2

Previously, we reported that the RBD of hnRNPA2 is sufficient for its function as a transcriptional coactivator of stress-target genes [19]. Currently, there is very little information about the structure of hnRNPA2 or its mode of interaction with RNA. To gain insight into the potential mechanism by which hnRNPA2 binds acetyl-CoA, a structural model of the RBDs of hnRNPA2 was generated using AMBER (see ‘Materials and Methods’ section). In the resulting model of the hnRNPA2: acetyl-CoA complex, the adenosine ring of acetyl-CoA (Figure 3e) is stabilized by *π*-stacking interactions with His 96 and Phe 12, whereas Arg 50 forms a hydrogen bond with the 5′-phosphate and Arg 48 participates in a bidentate hydrogen bond with two oxygen molecules of the pantothenate moiety. The RBDs of hnRNPA2 and hnRNPA1 are highly conserved, and the binding mode of hnRNPA2 and acetyl-CoA is analogous to that of adenine in the crystal structure of hnRNPA1 in complex with ssDNA (Figure 3e). The same binding residues appear to be conserved in hnRNPA2, suggesting that the conserved RBD has been repurposed to bind acetyl-CoA.

To test the proposed 3D model, we generated hnRNPA2 mutant proteins in which Arg residues were mutated either individually (R48T or R50T) or simultaneously (R48T/R50T) (coomassie blue staining for purified protein profile shown in Supplementary Figure S2C). The R48T/R50T double mutant exhibited only 10% of the KAT activity of the WT protein, whereas the R48T and R50T single mutants retained 25% and 50% of the KAT activity, respectively (Figure 3f). These results validated our structural prediction for acetyl-CoA binding, which also appears to be critical for KAT activity.

### HnRNPA2 acetylates histone H4 at K8 on target gene promoters

**Figure 4:**
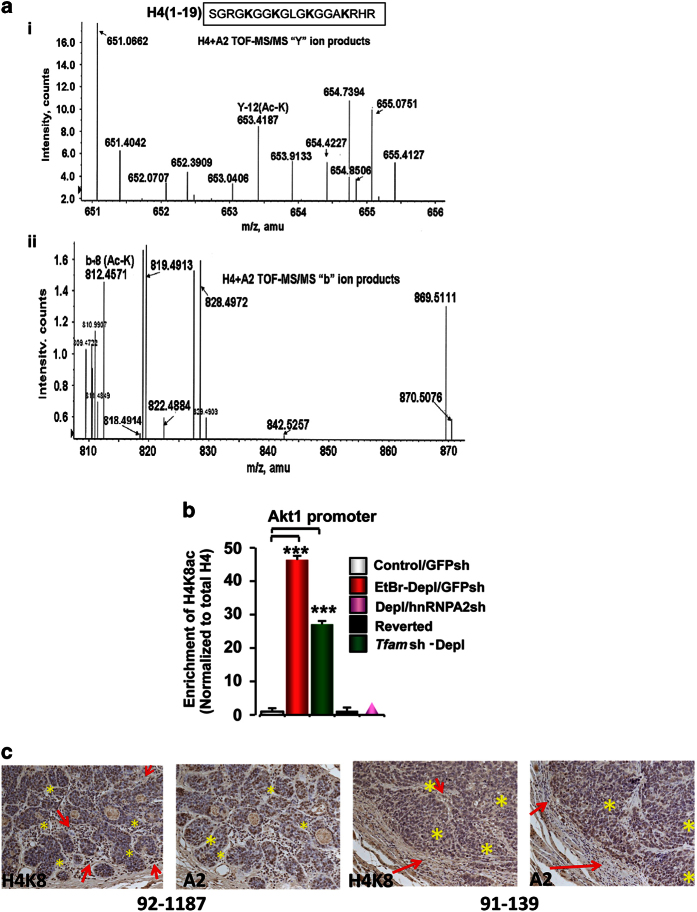
Site specificity of hnRNPA2-mediated H4 acetylation. (**a**) Identification of hnRNPA2-specific H4 acetylation sites by LC–MS/MS. The MS/MS spectrum of peptide ^1^SGRGKGGK_AC_GLGKGGAKRHR^19^ confirms K8 acetylation. (i) Y-ion products. The peak at *m*/*z* 653.41 (labeled Y-12) corresponds to acetyl lysine; (ii) b-ion products. The peak at *m*/*z* 812.45 (labeled b-8) corresponds to acetyl lysine. (**b**) ChIP assay showing enrichment of H4K8 acetylation at the *Akt1* promoter in control, PmtDNA-depl, PmtDNA-depl/hnRNPA2sh and reverted C2C12 cells. Antibodies specific to H4K8Ac, was used for immunoprecipitation. Rabbit IgG and H4K5ac antibody were used as negative antibody controls and β-actin promoter was used as a negative control for promoter DNA region (Supplementary Figure S3A–C). Data are represented as mean±s.d. (**c**) Immunohistochemistry of serial sections of esophageal squamous cell carcinoma from two representative patient samples stained with either acetyl-H4K8 or hnRNPA2 antibody as indicated in the figure and described in Supplementary Information. The * shows stained tumor cells and the red arrows indicate the stroma. Patient case numbers are indicated in the figure.

Lysine residues 5, 8, 12 and 16 of H4 are the principal sites of KAT-mediated acetylation [37] and are linked to transcription activation *in vivo*. LC–MS/MS analysis of H4 peptide (residues 1–19) in an *in vitro* acetylation reaction using acetyl-CoA and hnRNPA2, indicated that hnRNPA2 acetylates Lys 8 (Figure 4a). ChIP analysis using specific antibodies for H4K8ac at both the MtRS target promoters, *Akt1* (Figure 4b) and *Cathepsin L* (Supplementary Figure S3A) showed marked enrichment of H4 acetylation at Lys 8 in PmtDNA-depl cells, which was markedly reduced in PmtDNA-depl/hnRNPA2sh and reverted cells. At both *Akt1* and cathepsin L promoters, there was no preferential enrichment of H4K5 acetylation (data not shown), which further supports the possibility of K8 of H4 being the primary target site of hnRNPA2.

Previously, we reported that the β-actin promoter is not activated by MtRS [19]. The absence of selective enrichment of H4K8 acetylation of the β-actin promoter in PmtDNA-depl cells (Supplementary Figure S3C, left panel) further confirms that H4K8 acetylation is a selective response to MtRS. In addition, intronic regions of *Cathepsin L* and *Akt1* genes from control and PmtDNA-depl cells didn’t show differences (Supplementary Figure S3C, right panel).

Immunohistochemical analysis of sections from human esophageal squamous cell carcinoma tumors showed that hnRNPA2 is localized at elevated levels in the nuclei of cancer cells, whereas the level in normal basal cells in the squamous epithelium are negligible. We observed a tight overlap between immunostaining for H4K8 acetylation and hnRNPA2 in the nuclei of tumor cells (indicated by *), whereas the stroma (indicated by red arrows) showed no immunoreactivity to either hnRNPA2 or acetyl-H4K8 antibodies (Figure 4c). These results confirm that hnRNPA2 and hnRNPA2-mediated H4K8 acetylation that are components of MtRS are activated in human cancers.

**Figure 5:**
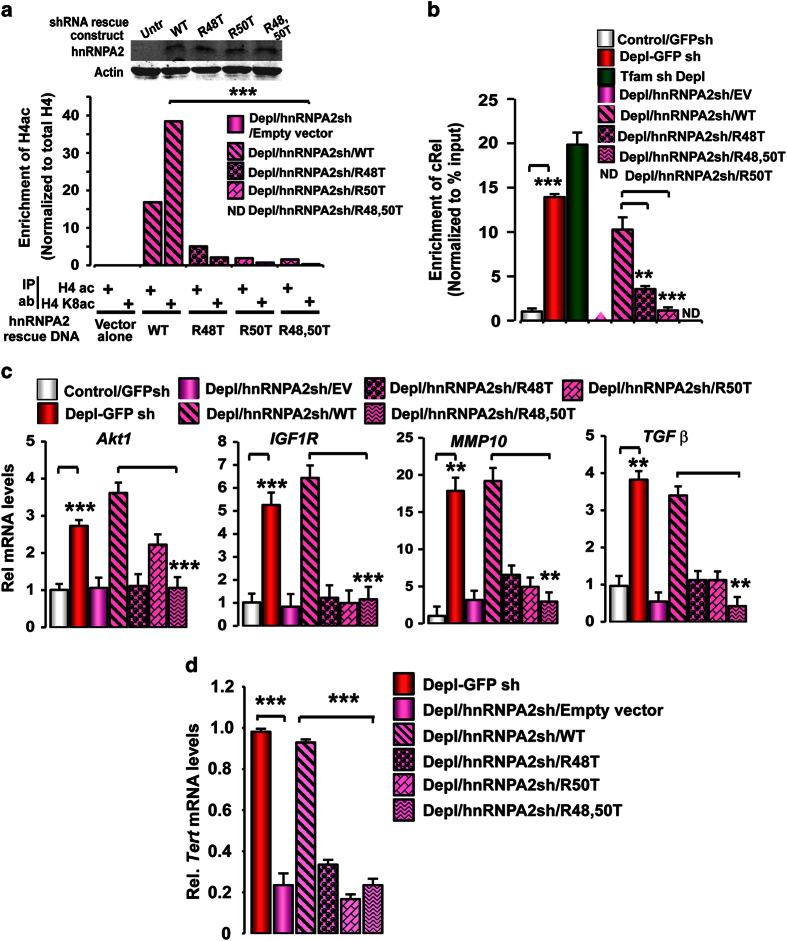
Functional role of hnRNPA2 KAT activity in stress-target gene transcription. (**a**, **b**) Involvement of the hnRNPA2 KAT residues in H4 acetylation at *Akt1* promoter detected by ChIP assay in PmtDNA-depl/hnRNPA2sh cells ectopically expressing either the pMXs vector alone or hnRNPA2 WT, R48T, R50T and R48T/R50T mutants (Supplementary Figure S3D). Immunoblot (inset) showing similar expression levels of all hnRNPA2shRNA rescue constructs in PmtDNA-depl/hnRNPA2sh cells. (**b**) Association of stress-target factor, cRel at the Akt1 promoter in control, PmtDNA-depl, PmtDNA-depl/hnRNPA2sh and in PmtDNA-depl/hnRNPA2sh cells ectopically expressing hnRNPA2 WT and KAT mutants. (**c**) Transcript levels of target genes in control, PmtDNA-depl, PmtDNA-depl/hnRNPA2sh and PmtDNA-depl/hnRNPA2sh expressing KAT mutants in C2C12 cells (Supplementary Figures S3D and S4; Supplementary Table S1). (**d**) *Tert* transcript levels in control, PmtDNA-depl, PmtDNA-depl/hnRNPA2sh and PmtDNA-depl/hnRNPA2sh cells expressing WT and KAT mutant cells. Data are represented as mean±s.d.

To test if the amino acid residues R48 and R50 of hnRNPA2 have a role in H4K8 acetylation, we generated PmtDNA-depl/hnRNPA2sh cell lines ectopically expressing either hnRNPA2 WT or mutant proteins (R48T, R50T or R48T/R50T) by introducing degenerate codons within the shRNA target sites of cDNAs (see ‘Materials and Methods’ section). The immunoblot presented in Figure 5a (inset, top panel) shows hnRNPA2 protein levels were comparable among cells expressing shRNA rescue constructs. As expected, we did not observe detectable H4K8 acetylation at the target gene promoter (Figure 5a) in PmtDNA-depl/hnRNPA2sh cells. Cells reconstituted with the WT hnRNPA2 showed ~40-fold enrichment in H4K8-specific acetylation promoter DNA, whereas cells reconstituted with the hnRNPA2 mutants (Arg 48/50 T) had undetectable H4K8 acetylation. In these PmtDNA-depl/hnRNPA2sh cells, rescuing with WT, but not the Arg mutant hnRNPA2 restored H4K8 acetylation at the stress-target promoters (Figure 5a; Supplementary Figure S3D). These results suggest that Arg 48 and Arg 50 residues are essential for hnRNPA2 functions in modulating histone acetylation *in vivo* at the target gene promoters.

### HnRNPA2-mediated H4 acetylation is important for its transcription coactivator function

Overexpression of WT hnRNPA2 in PmtDNA-depl/hnRNPA2sh cells resulted in ~fivefold activation of the *Cathepsin L* promoter, whereas expression of the KAT domain mutants (R48T, R50T and R48T/R50T) abrogated this activation (Supplementary Figure S3E). We previously reported that the occupancy of the stress-activated transcription factor cRel:p50 along with the other factors on the stress-target gene promoter is essential for target gene activation. Furthermore, this recruitment of cRel is part of MtRS and dependent on hnRNPA2. Here, we observed that the recruitment of cRel to the *Akt1* promoter was markedly diminished in PmtDNA-depl/hnRNPA2sh cells expressing the KAT mutants (Figure 5b). We observed that the increased transcription of a panel of previously reported mitochondrial stress-target genes was dependent on the hnRNPA2 KAT activity. Ectopically expressing WT hnRNPA2 in PmtDNA-depl/hnRNPA2sh cells rescued the transcript levels to that seen in PmtDNA-depl cells, whereas the target gene transcription was markedly impaired in cells expressing hnRNPA2 KAT mutant proteins (Figure 5c and d).

**Figure 6:**
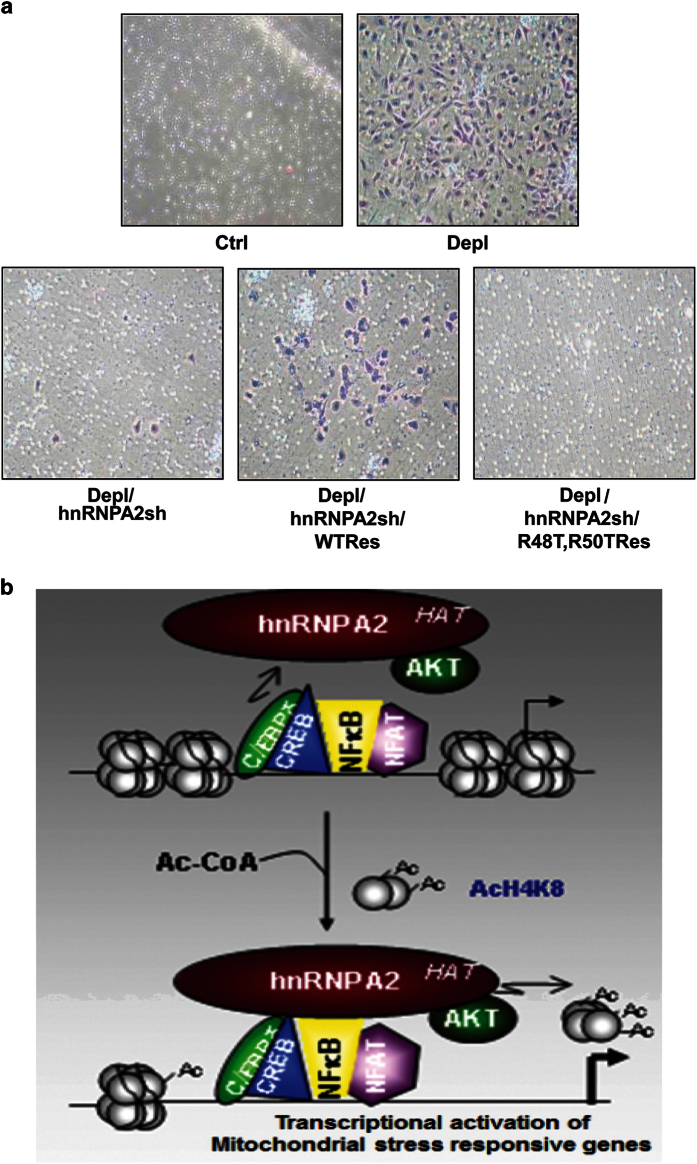
HnRNPA2 KAT activity is essential for the mtDNA depletion-induced invasive phenotype. (**a**) Matrigel invasion assay in control, PmtDNA-depleted, PmtDNA-depl/hnRNPA2sh cells and PmtDNA-depl/hnRNPA2sh cells expressing either WT or KAT mutant hnRNPA2. A representative image from three independent experiments is presented. (**b**) Schematic showing hnRNPA2 acetylates histone H4 at the promoter DNA of the mitochondrial stress-target genes. Akt activated by MtRS phosphorylates hnRNPA2 and augments its KAT activity. Histone acetylation by hnRNPA2 provides access to stress-activated transcription factors NFκB, C/EBPδ, CREB and NFAT to the promoter DNA, resulting in transcriptional activation of the stress-target genes.

We tested the functional consequences of the KAT activity of hnRNPA2 on the global oncogene expression pattern using the mouse cancer pathway finder RT^2^ profiler array, which profiles the expression of 84 genes representative of 9 different biological pathways involved in tumorigenesis (SA Biosciences, Qiagen, Frederick, MD, USA). The RT^2^ profiler analysis shows a number of genes involved in oncogenic processes were altered in PmtDNA-depl C2C12 cells and were affected by hnRNPA2 KAT mutations (Supplementary Table S1; Supplementary Figure S4). In accordance with these observations, the acquired invasive potential of PmtDNA-depl C2C12 cells was lost in cells expressing hnRNPA2 KAT mutants (Figure 6a). These results establish a crucial link between hnRNPA2 KAT activity and its function as a transcriptional coactivator in response to PmtDNA depletion as outlined in Figure 6b.

## Discussion

Previously, we showed that activation of hnRNPA2 is essential for the propagation of MtRS and its silencing abrogated stress response, leading to reversal of the invasive and proliferative phenotype of cells [2, 13–15, 17, 19–21]. Specifically, we showed that hnRNPA2 acts as a transcription coactivator by binding to the 5′ upstream promoter sites mostly through protein–protein interaction with other DNA binding transcription factors. In this study, we show that hnRNPA2 is a novel KAT protein, which modulates the expression of stress-response genes by H4K8 acetylation at the promoter sites. We also show that mitochondrial dysfunction in immortalized cells induces telomerase activation, which probably prevents cells from undergoing senescence. In addition, expression of *Tert* and *Terc* genes are also modulated by MtRS-activated hnRNPA2.

PmtDNA-depl cells appear to evade senescence by MtRS-mediated activation of telomerase. HnRNPA2 and Akt, two important components of MtRS, have a causal role in transcriptional upregulation of telomerase components, *Tert* and *Terc. In silico* analysis indeed shows the presence of the four factor binding sites (CREB, NFATc, cRel;p50 and C/EBPδ) immediately upstream of transcription start sites. These factor binding foot-prints are similar to those shown for stress-target *Akt1* and *RyR1* genes (Figure 1). Reactivation of telomerase in PmtDNA-depl cells is consistent with our previous report [38] showing that these cells are resistant to apoptosis.

The KAT activity of hnRNPA2 is demonstrated by the following criteria: first, purified recombinant hnRNPA2 and its RBD show KAT activity with both H4 and also with purified nucleosomes. Second, purified acetyl-CoA binding site mutant hnRNPA2 protein showed diminished activity *in vitro* and *in vivo* in reconstituted cells. Third, shRNA-mediated knockdown of hnRNPA2 caused diminished H4K8 acetylation followed by low transcriptional activation of target genes. Finally, *in vitro* translated hnRNPA2 programmed with WT cDNA showed KAT activity. These results dispel any possibility of a contaminating protein catalyzing the KAT activity. Finally, the KAT activity of hnRNPA2 is effectively inhibited by garcinol/isogarcinol and curcumarin derivatives that are known KAT inhibitors [35, 36].

The property of hnRNPA2 as a KAT is similar to other transcriptional coactivators that remodel chromatin DNA by histone acetylation (Figure 6b). Interestingly, our observation that Akt phosphorylation augments its histone acetylation function is in agreement with a recent study showing the requirement for Akt in modulating histone acetylation status in tumor cells exhibiting metabolic defects [39]. Within the limits of the computational methods used to derive the common structural elements between the adenine moiety of DNA and acetyl-CoA, we propose a structural model for the binding of acetyl-CoA to hnRNPA2 (Figure 3e). This model also relies on the single strand DNA/RNA binding mode of the highly similar hnRNPA1 protein. Importantly, despite the high conservation of RBDs, hnRNPA1 did not show any measurable KAT activity (Supplementary Figure S2G). It is likely that the more variable sequence region of hnRNPA2, upstream of the RBD is necessary for the KAT activity. Interestingly, our data show that substitutions at Arg 48 and Arg 50 residues result in a loss of histone acetylation function of hnRNPA2. It could be argued that hnRNPA2 is a chaperone protein and modulates histone acetylation by recruiting other acetyltransferase(s) as shown for PGC-1α [40]. It must be noted however that promoter pull-down analysis using mitochondrial stress-target gene promoters did not detect any other known KAT proteins [19]. Our findings on the increased hnRNPA2-mediated histone acetylation under mitochondrial stress conditions are in agreement with previous reports showing increased global histone acetylation in mtDNA-depleted cells, or cells carrying heteroplasmic mutations [41, 42]. Although not shown, partial mtDNA depletion as used in this study either by chemical (EtBr) or genetic (TFAM shRNA expression) approaches do not diminish cellular Acetyl-CoA levels. Instead, Acetyl-CoA levels are increased modestly or significantly in these cells, supporting increased acetylation.

Others and we have shown that mitochondrial stress induces global alterations in nuclear gene expression pattern [41–44], and increased histone acetylation by altered metabolism and increased NADH pool [45–47]. Here, we provide a mechanistic link mediated via hnRNPA2 demonstrating that MtRS regulates epigenetic events, by histone acetylation on the target genes. These findings are particularly significant in the context of cancer progression where reduction in MtDNA copy number has been widely observed in tumors. Moreover, hnRNPA2 modulates different aspects of cancer cell metabolism, invasion and cancer progression by alternative splicing and transcriptional activation of oncogenes [22, 23, 48–50], and this study suggests that hnRNPA2 possibly has a global role in gene activation by epigenetic modifications. Our findings highlight the significance of mitochondrial dysfunction-induced MtRS as a common epigenetic link for nuclear gene expression, which may be important in cancer and other pathologies.

## Materials and Methods

### Cell lines

Murine skeletal myoblasts C2C12 cells were purchased from ATCC (CRL 1772) and cultured in DMEM with 10% fetal bovine serum. MtDNA was partially depleted (70%) using ethidium bromide (100 ng ml^−1^) or 2,3′-dideoxycytidine (ddC; 10 μm, 120 h) or *Tfam* shRNA as previously described [2, 14]. EtBr and ddC were washed out from the cell growth medium for at least 2–3 passages before being used for experiments. These chemicals were not present when the experiments were performed. In both methods (Tfam shRNA or EtBR treatment), we have used about 80% mtDNA depletion as the starting point. MtDNA-depleted C2C12 cells were supplemented with 1 mm pyruvate and 50 μg ml^−1^ uridine. Reverted cells represent PmtDNA-depleted cells grown subsequently for 30 passages in the absence of EtBr, for reversing the MtDNA content to 80% of control cells.

Three independent shRNA constructs targeting hnRNPA2 were validated in preliminary transient transfection experiments as previously described [19]. MtDNA-depleted C2C12 cells stably expressing either hnRNPA2 or GFP (negative control) shRNA (cloned in pLKO.1 vector) were generated. Over 85% of the hnRNPA2 mRNA and protein has been silenced. For hnRNPA2 rescue experiments in C2C12 cells, the hnRNPA2 mutant constructs used in this study were subcloned in pMXs vector (kind gift from Russ Carstens, UPENN) by Gene Pass (Nashville, TN, USA). HnRNPA2shRNA rescue cell lines were generated by ectopically expressing hnRNPA2 with conservative mutations in the shRNA target regions. For generation of stably expressing shRNA rescue hnRNPA2 KAT mutants, PmtDNA-depl/hnRNPA2sh expressing cells were transduced with either pMXS-IRES- Puro- EGFP empty vector or KAT mutant cDNAs. Rescue of hnRNPA2 expression in PmtDNA-depl/hnRNPA2sh cells was confirmed by western immunoblot.

Five shRNA constructs were used for *Tfam* and three shRNA constructs were used for hnRNPA2. ShRNA targeting GFP was used as control. For Tfam silencing experiments, Tfam and GFP shRNAs cloned in pLKO.1 lentiviral vectors were used. Five independent Tfam shRNA constructs were used for the initial screening experiments. Cells were transduced using 48 and 72 h viral supernatant and shRNA expressing cells were selected by puromycin (2 μg ml^−1^) selection for about five cell passages. After antibiotic selection, Tfam mRNA levels were reduced by 80% and depending on the cell type, it required another 5–7 passages for mtDNA content to be reduced by 80%. Cells expressing Tfam shRNA were maintained in medium with added uridine (50 μg ml^−1^) and sodium pyruvate (1 mm).

### Plasmids

Full-length HnRNPA2 (human) cDNA cloned in pET 28a (+) vector with N-terminal ×6 His tag was a gift from Gideon Dreyfuss (University of Pennsylvania). RBD (residues 1–180) and glycine-rich domain (residues 180–341) were PCR amplified and cloned in the same vector. Full-length ×6 His-hnRNPA2/ pET 28a(+) with Arg 48, Arg 50 mutations and ×6-hnRNPA2-RBD/pET 28a(+) with Phe 12 and His 96 mutations were generated using the Quick Change Lightning site-directed mutagenesis kit (Agilent technologies, Wilmington, DE, USA).

### Expression and affinity purification of His-hnRNPA2

Full-length and mutant hnRNPA2 cDNAs cloned in pET 28a(+) bacterial expression vector, were transformed in BL21 bacterial cells. Protein expression was induced by treating 10 l of exponentially growing culture with 1 mm isopropyl thiogalactoside (IPTG) for 3 h at 30 °C. After incubation bacteria was lysed by sonication in buffer containing 50 mm NaH_2_PO_4_, 500 mm NaCl, 5% glycerol, 50 mm imidazole, pH 8 in the presence of lysozyme (1 mg ml^−1^) and DNase I (5 μg ml^−1^). The lysate was clarified by centrifugation at 14 000 r.p.m. for 20 min at 4 °C. His-tagged proteins were purified from the clarified lysates in two steps using a combination of Nickel affinity chromatography and ion exchange chromatography on an AKTA FPLC system. In the first step, the lysate was loaded on a HisTrap FF column (GE) pre-equilibrated with binding buffer (500 mm NaCl, 50 mm imidazole, 10% glycerol, pH 8). His-tagged proteins were eluted using a linear gradient of 0–100% elution buffer (500 mm NaCl, 500 mm imidazole, 10% glycerol, pH 8) at a flow rate of 0.5 ml min^−1^. Fractions containing hnRNPA2 from this step were pooled and dialyzed to remove imidazole and change buffer for ion exchange chromatography using Centricon concentrators. Full-length hnRNPA2 was further purified on a MonoS column, whereas RBD was purified on MonoQ column. The equilibration buffer was 20 mm sodium phosphate (pH 8). Elution was carried out at flow rate of 1 ml min^−1^ using a linear gradient of 0–100% elution buffer. Elution was carried out at flow rate 1 ml min^−1^ using a linear gradient of 0–100% elution buffer (20 mm sodium phosphate, pH 8 and 1m NaCl). HnRNPA2 fractions were dialyzed, concentrated and flash frozen in liquid nitrogen before storing at −80 °C. The protein yield from a 10 l bacterial culture was ~25 mg. Interestingly, similar to other HAT proteins recombinant hnRNPA2 was highly toxic to *E. coli*, which could account for the low yield of the full-length protein besause of indiscriminate acetylation of host proteins.

### Quantification of MtDNA

Analysis of MtDNA content from cellular total DNA was performed using real time qPCR as described before [14]. Copy number of MtDNA coded gene (CcO I) was normalized to nuclear single copy gene (CcO IVi1).

### *Invitro* kinase assay

The *in vitro* kinase assay was carried out as detailed previously [21]. A total of 0.1 μg of Recombinant Akt (Millipore, Billerica, MA, USA) was incubated for 30 min at 37 °C with purified His-hnRNPA2 (0.5 μg) in a buffer containing 10 mm HEPES (pH 7.4), 1 mm MgCl_2_ and 1 mm MnCl_2_ in the presence of 10 μCi [γ-32 P]ATP.

### KAT assays

Filter-binding KAT assays were performed as previously described [51]. Affinity-purified recombinant hnRNPA2 proteins (100 nm) were incubated at 30 °C for 20 min with either 50 μm synthetic histone amino terminal peptides H4 (residues 1–19; active motif) or 5 μg of HeLa mononucleosomes (EpiCypher, The Woodlands, TX, USA, Cat#16-0002) in the presence of either 50 μm ^14^C acetyl-CoA (American Radiolabeled Chemicals, Saint Louis, MO, USA) in a 30 μl reaction volume. Reaction products were spotted on P-81 filters (Whatman), air-dried and washed extensively in 0.2m sodium carbonate buffer and scintillation counts were taken.

For the KAT assays with the inhibitors, CTK7A is a water soluble compound and LTK14, garcinol and isogarcinol are solubilized in DMSO. DMSO alone was used in control assays run alongside. Notably, DMSO alone did not show any effect on the HAT activity of hnRNPA2 protein (data not shown).

### Identification of lysine acetylation by LC–MS/MS analysis

LC–MS/MS was carried out to detect H4 acetylation by hnRNPA2. Briefly, HAT reaction for identifying acetylation site LC–MS/MS was carried out as detailed above. The reverse-phase nanobore liquid chromatography was performed on an Agilent LC 1100 (Agilent) fitted with an auto sampler (G1377A), a cap pump (G1376A), a nano pump (G2225A) and a 1100 control module (G1323) that was connected to a NanoSpray QSTAR-XL mass spectrometer (Applied Biosystems/MDS Sciex, Framingham, MA, USA). Briefly, 4 μl of acetylation reaction mixture was loaded onto the Precolumn, ZORBOX300SB C18 column (0.3×5 mm) and washed with formic acid/H_2_O (0.1/99.9, by v/v) at a flow rate of 5 μl min^−1^ (capillary pump). The eluent was transferred to the separation column, ZORBOX C18 column (0.075×150 mm) from Agilent. Gradient elution (flow rate: 0.3 μl min^−1^) from this column consisted of a 5 min initial isocratic elution with 0% B, a linear gradient 0–70% B in 35 min, a 10 min isocratic elution 70% B and a linear gradient 70–0% B in 5 min. The eluent was directly introduced into NanoSpray QSTAR-XL mass spectrometer (Applied Biosystems/MDS Sciex). Spray capillary was a PicoTip emitter with ID 15 μm (New Objective, Woburn, MA, USA). The MS instrument was operated with the following setting: curtain gas 20 l min^−1^, spray voltage 2.5 kV, declustering potential 60 V, focusing potential 220 V, collision gas 5 l min^−1^ and collision energy optimized automatically. TOF-MS and TOF-MS/MS spectra were acquired in the information dependent acquisition method performed with Analyst QS1.1 software provided by Applied Biosystems/MDS Sciex. Sequencing analysis was done by manual method based on Bioanalyst Software 1.1.5 *de novo* peptide sequencing provided by Applied Biosystems/MDS Sciex.

### Modeling of hnRNPA2 in complex with acetyl-CoA

The hnRNPA2 model has a basic groove postulated to bind acetyl-CoA (green), with a clear binding pocket that accommodates the adenosine ring and specific hydrogen bond interactions with Arg 48 and Arg 50 [51]. The electrostatic surface potential map was generated with the Delphi software package24 and is colored from −7 kTe-1 (blue) to +7 kTe-1 (red)

The protein sequence corresponding to the RBD of hnRNPA2 was submitted to the Phyre2 server [PMID 19247286]. Phyre2 uses the hidden Markov method to generate alignments of a submitted protein sequence against proteins with structures available in the Protein Data Bank [PMID 15531603]. The resulting alignments are then used to produce a homology-based model of the query sequence to predict its three-dimensional structure. The model is scored as accurate when over 90% of the submitted residues are modeled at greater than 90% confidence [PMID 19247286]. The resulting model for the RBD of hnRNPA2 was then used in flexible docking of acetyl-CoA. A docking calculation is a computational search for the binding conformations or poses of a given ligand that have the highest complementarity to the protein surface and lowest calculated energy score using an interaction force field that includes van der Waals and electrostatic terms. UCSF DOCK utilizes the ‘anchor and grow’ method, whereby a scaffold or anchor component of the ligand is identified and then rotatable bonds are searched and added on during the growth phase [PMID: 19369428]. Docking spheres for hnRNPA2 were generated using all surface points, with maximum and minimum sphere radii of 4 Å and 1.4 Å, respectively. An energy grid with 0.3 Å spacing was calculated, using attractive and repulsive exponents of 6 and 9, respectively. A dielectric factor of 4 and a bump overlap of 0.75 Å was utilized. The acetyl-CoA molecule was charged using AmberTools and all docking calculations were performed with UCSF DOCK [PMID: 19369428]. The final docked pose for acetyl-CoA had a total grid score of −81.9 kcal mol^−1^ with the electrostatic and van der Waals components being −70.5 kcal mol^−1^ and −11.4 kcal mol^−1^, respectively.

### ChIP analysis

ChIP assays were performed as previously described [19]. Immunoprecipitation of cross linked chromatin–protein complexes were carried out using following antibodies: anti-hnRNPA2 (Santa Cruz, Dallas, TX, USA), anti-hyperacetylated H4 histone, anti-acetylated histone H4 at lysine 8 or 5 (Upstate Biotechnology, Lake Placid, NY, USA). Real time PCR analysis was done using primer sets to Cathepsin L, RyR1 and Akt1 promoters. Rabbit preimmune serum, H4K5ac antibody were used as negative antibody controls and β-actin promoter, which is non-responsive to mitochondrial stress, was used as a negative control for promoter DNA region. ChIP values were normalized to either the total input DNA or total histone H4 levels, as indicated in figures, to adjust for differences in chromatin DNA or total H4 levels. Real time PCR were carried out in triplicates from at least three independent experiments.

### Matrigel invasion assay

*In vitro* invasion assays were carried out as described before [19]. Cells (5×10^4^) in growth medium were seeded on a Matrigel-coated Boyden chamber. After 24 h, cells that invaded the Matrigel were stained with hematoxylin-eosin and observed under bright field microscope.

### Cellular respiration

Oxygen consumption and extracellular rate of acidification was carried out in a XF24 Seahorse Analyzer (Seahorse Bioscience, Billerica, MA, USA) using 5×10^4^ cells. For assessing ATP-coupled respiration, Oligomycin (2 μg ml^−1^), DNP (75 μm) and Rotenone (1 μm) were added sequentially.

### Immunohistochemistry

Immunostaining of human esophageal cancer sections was done using Vectastain ABC kit (Vector laboratories, Burlingame, CA, USA) according to manufacturer’s instructions. Briefly, sections were deparaffinized and incubated in blocking buffer for 1 h at 37 °C. Incubation with primary antibodies (acetylated-H4K8 or hnRNPA2) was carried out overnight at 4 °C. Biotinylated secondary IgG incubation was then carried out at 37 °C and the signal was developed using the DAP peroxidase staining kit (Vector laboratories).

### Statistical analysis

All data are representative of at least three independent experiments. Statistical significance was determined using student’s *t*-test. *P*-values <0.05 (*) and ⩽0.005 (**) were considered statistically significant, and *P*-values <0.001 (***) were highly significant.

## References

[bib1] Greaves LC , Reeve AK , Taylor RW , Turnbull DM . Mitochondrial DNA and disease. J Pathol 2012; 226: 274–286. 21989606 10.1002/path.3028

[bib2] Biswas G , Adebanjo OA , Freedman BD et al. Retrograde Ca2+ signaling in C2C12 skeletal myocytes in response to mitochondrial genetic and metabolic stress: a novel mode of inter-organelle crosstalk. EMBO J 1999; 18: 522–533. 9927412 10.1093/emboj/18.3.522 PMC1171145

[bib3] Butow RA , Avadhani NG . Mitochondrial signaling: the retrograde response. Mol Cell 2004; 14: 1–15. 15068799 10.1016/s1097-2765(04)00179-0

[bib4] Chandel NS , Maltepe E , Goldwasser E et al. Mitochondrial reactive oxygen species trigger hypoxia-induced transcription. Proc Natl Acad Sci USA 1998; 95: 11715–11720. 9751731 10.1073/pnas.95.20.11715 PMC21706

[bib5] Irrcher I , Ljubicic V , Hood DA . Interactions between ROS and AMP kinase activity in the regulation of PGC-1alpha transcription in skeletal muscle cells. Am J Physiol Cell Physiol 2009; 296: C116–C123. 19005163 10.1152/ajpcell.00267.2007

[bib6] Chae S , Ahn BY , Byun K et al. A systems approach for decoding mitochondrial retrograde signaling pathways. Sci Signal 2013; 6: rs4. 23443683 10.1126/scisignal.2003266

[bib7] Arnould T , Vankoningsloo S , Renard P et al. CREB activation induced by mitochondrial dysfunction is a new signaling pathway that impairs cell proliferation. EMBO J 2002; 21: 53–63. 11782425 10.1093/emboj/21.1.53 PMC125809

[bib8] DiMauro S , Schon EA . Mitochondrial respiratory-chain diseases. N Engl J Med 2003; 348: 2656–2668. 12826641 10.1056/NEJMra022567

[bib9] Raimundo N , Song L , Shutt TE et al. Mitochondrial stress engages E2F1 apoptotic signaling to cause deafness. Cell 2012; 148: 716–726. 22341444 10.1016/j.cell.2011.12.027 PMC3285425

[bib10] Ryan MT , Hoogenraad NJ . Mitochondrial-nuclear communications. Annu Rev Biochem 2007; 76: 701–722. 17227225 10.1146/annurev.biochem.76.052305.091720

[bib11] Wallace DC . Mitochondria and cancer. Nat Rev Cancer 2012; 12: 685–698. 23001348 10.1038/nrc3365 PMC4371788

[bib12] Guha M , Avadhani NG . Mitochondrial retrograde signaling at the crossroads of tumor bioenergetics, genetics and epigenetics. Mitochondrion 2013; 13: 577–591. 24004957 10.1016/j.mito.2013.08.007 PMC3832239

[bib13] Amuthan G , Biswas G , Zhang SY et al. Mitochondria-to-nucleus stress signaling induces phenotypic changes, tumor progression and cell invasion. EMBO J 2001; 20: 1910–1920. 11296224 10.1093/emboj/20.8.1910 PMC125420

[bib14] Guha M , Srinivasan S , Ruthel G et al. Mitochondrial retrograde signaling induces epithelial–mesenchymal transition and generates breast cancer stem cells. Oncogene 2014; 33: 5238–5250. 24186204 10.1038/onc.2013.467 PMC4921233

[bib15] Tang W , Chowdhury AR , Guha M et al. Silencing of IkBbeta mRNA causes disruption of mitochondrial retrograde signaling and suppression of tumor growth in vivo. Carcinogenesis 2012; 33: 1762–1768. 22637744 10.1093/carcin/bgs190 PMC3514893

[bib16] Woo DK , Green PD , Santos JH et al. Mitochondrial genome instability and ROS enhance intestinal tumorigenesis in APC(Min/+) mice. Am J Pathol 2012; 180: 24–31. 22056359 10.1016/j.ajpath.2011.10.003 PMC3338350

[bib17] Amuthan G , Biswas G , Ananadatheerthavarada HK et al. Mitochondrial stress-induced calcium signaling, phenotypic changes and invasive behavior in human lung carcinoma A549 cells. Oncogene 2002; 21: 7839–7849. 12420221 10.1038/sj.onc.1205983

[bib18] Guha M , Srinivasan S , Biswas G , Avadhani NG . Activation of a novel calcineurin-mediated insulin-like growth factor-1 receptor pathway, altered metabolism, and tumor cell invasion in cells subjected to mitochondrial respiratory stress. J Biol Chem 2007; 282: 14536–14546. 17355970 10.1074/jbc.M611693200 PMC3800738

[bib19] Guha M , Pan H , Fang JK , Avadhani NG . Heterogeneous nuclear ribonucleoprotein A2 is a common transcriptional coactivator in the nuclear transcription response to mitochondrial respiratory stress. Mol Biol Cell 2009; 20: 4107–4119. 19641020 10.1091/mbc.E09-04-0296 PMC2743628

[bib20] Guha M , Tang W , Sondheimer N , Avadhani NG . Role of calcineurin, hnRNPA2 and Akt in mitochondrial respiratory stress-mediated transcription activation of nuclear gene targets. Biochim Biophys Acta 2010; 1797: 1055–1065. 20153290 10.1016/j.bbabio.2010.02.008 PMC2891149

[bib21] Guha M , Fang JK , Monks R , Birnbaum MJ , Avadhani NG . Activation of Akt is essential for the propagation of mitochondrial respiratory stress signaling and activation of the transcriptional coactivator heterogeneous ribonucleoprotein A2. Mol Biol Cell 2010; 21: 3578–3589. 20719961 10.1091/mbc.E10-03-0192 PMC2954122

[bib22] Golan-Gerstl R , Cohen M , Shilo A et al. Splicing factor hnRNP A2/B1 regulates tumor suppressor gene splicing and is an oncogenic driver in glioblastoma. Cancer Res 2011; 71: 4464–4472. 21586613 10.1158/0008-5472.CAN-10-4410

[bib23] Moran-Jones K , Grindlay J , Jones M , Smith R , Norman JC . hnRNP A2 regulates alternative mRNA splicing of TP53INP2 to control invasive cell migration. Cancer Res 2009; 69: 9219–9227. 19934309 10.1158/0008-5472.CAN-09-1852 PMC6485436

[bib24] Grunstein M . Histone acetylation in chromatin structure and transcription. Nature 1997; 389: 349–352. 9311776 10.1038/38664

[bib25] Xu CR , Cole PA , Meyers DJ et al. Chromatin ‘prepattern’ and histone modifiers in a fate choice for liver and pancreas. Science 2011; 332: 963–966. 21596989 10.1126/science.1202845 PMC3128430

[bib26] Brown CE , Lechner T , Howe L , Workman JL . The many HATs of transcription coactivators. Trends Biochem Sci 2000; 25: 15–19. 10637607 10.1016/s0968-0004(99)01516-9

[bib27] Du X , Shen J , Kugan N et al. Telomere shortening exposes functions for the mouse Werner and Bloom syndrome genes. Mol Cell Biol 2004; 24: 8437–8446. 15367665 10.1128/MCB.24.19.8437-8446.2004 PMC516757

[bib28] Thompson PR , Wang D , Wang L et al. Regulation of the p300 HAT domain via a novel activation loop. Nat Struct Mol Biol 2004; 11: 308–315. 15004546 10.1038/nsmb740

[bib29] Yuan H , Rossetto D , Mellert H et al. MYST protein acetyltransferase activity requires active site lysine autoacetylation. EMBO J 2012; 31: 58–70. 22020126 10.1038/emboj.2011.382 PMC3252582

[bib30] Sun B , Guo S , Tang Q et al. Regulation of the histone acetyltransferase activity of hMOF via autoacetylation of Lys274. Cell Res 2011; 21: 1262–1266. 21691301 10.1038/cr.2011.105 PMC3193475

[bib31] Albaugh BN , Arnold KM , Lee S , Denu JM . Autoacetylation of the histone acetyltransferase Rtt109. J Biol Chem 2011; 286: 24694–24701. 21606491 10.1074/jbc.M111.251579 PMC3137045

[bib32] Yang C , Wu J , Sinha SH , Neveu JM , Zheng YG . Autoacetylation of the MYST Lysine Acetyltransferase MOF Protein. J Biol Chem 2012; 287: 34917–34926. 22918831 10.1074/jbc.M112.359356 PMC3471714

[bib33] Huang WC , Chen CC . Akt phosphorylation of p300 at Ser-1834 is essential for its histone acetyltransferase and transcriptional activity. Mol Cell Biol 2005; 25: 6592–6602. 16024795 10.1128/MCB.25.15.6592-6602.2005 PMC1190347

[bib34] Arif M , Vedamurthy BM , Choudhari R et al. Nitric oxide-mediated histone hyperacetylation in oral cancer: target for a water-soluble HAT inhibitor, CTK7A. Chem Biol 2010; 17: 903–913. 20797619 10.1016/j.chembiol.2010.06.014

[bib35] Mantelingu K , Reddy BA , Swaminathan V et al. Specific inhibition of p300-HAT alters global gene expression and represses HIV replication. Chem Biol 2007; 14: 645–657. 17584612 10.1016/j.chembiol.2007.04.011

[bib36] Brownell JE , Zhou J , Ranalli T et al. Tetrahymena histone acetyltransferase A: a homolog to yeast Gcn5p linking histone acetylation to gene activation. Cell 1996; 84: 843–851. 8601308 10.1016/s0092-8674(00)81063-6

[bib37] Biswas G , Anandatheerthavarada HK , Avadhani NG . Mechanism of mitochondrial stress-induced resistance to apoptosis in mitochondrial DNA-depleted C2C12 myocytes. Cell Death Differ 2005; 12: 266–278. 15650755 10.1038/sj.cdd.4401553

[bib38] Lee JV , Carrer A , Shah S et al. Akt-dependent metabolic reprogramming regulates tumor cell histone acetylation. Cell Metab 2014; 20: 306–319. 24998913 10.1016/j.cmet.2014.06.004 PMC4151270

[bib39] Wallberg AE , Yamamura S , Malik S , Spiegelman BM , Roeder RG . Coordination of p300-mediated chromatin remodeling and TRAP/mediator function through coactivator PGC-1alpha. Mol Cell 2003; 12: 1137–1149. 14636573 10.1016/s1097-2765(03)00391-5

[bib40] Guantes R , Rastrojo A , Neves R et al. Global variability in gene expression and alternative splicing is modulated by mitochondrial content. Genome Res 2015; 25: 633–644. 25800673 10.1101/gr.178426.114 PMC4417112

[bib41] Picard M , Zhang J , Hancock S et al. Progressive increase in mtDNA 3243A>G heteroplasmy causes abrupt transcriptional reprogramming. Proc Natl Acad Sci USA 2014; 111: E4033–E4042. 25192935 10.1073/pnas.1414028111 PMC4183335

[bib42] Biswas G , Tang W , Sondheimer N et al. A distinctive physiological role for Ikappa Bbeta in the propagation of mitochondrial respiratory stress signaling. J Biol Chem 2008. 10.1074/jbc.M710481200 PMC2335355 18272519

[bib43] Srinivasan S , Guha M , Dong DW et al. Disruption of cytochrome c oxidase function induces Warburg effect and metabolic reprogramming. Oncogene 2016; 35: 1585–1595. 26148236 10.1038/onc.2015.227 PMC4703574

[bib44] Friis RM , Glaves JP , Huan T et al. Rewiring AMPK and mitochondrial retrograde signaling for metabolic control of aging and histone acetylation in respiratory-defective cells. Cell Rep 2014; 7: 565–574. 24726357 10.1016/j.celrep.2014.03.029

[bib45] Lu C , Thompson CB . Metabolic regulation of epigenetics. Cell Metab 2012; 16: 9–17. 22768835 10.1016/j.cmet.2012.06.001 PMC3392647

[bib46] Schroeder EA , Raimundo N , Shadel GS . Epigenetic silencing mediates mitochondria stress-induced longevity. Cell Metab 2013; 17: 954–964. 23747251 10.1016/j.cmet.2013.04.003 PMC3694503

[bib47] David CJ , Chen M , Assanah M , Canoll P , Manley JL . HnRNP proteins controlled by c-Myc deregulate pyruvate kinase mRNA splicing in cancer. Nature 2010; 463: 364–368. 20010808 10.1038/nature08697 PMC2950088

[bib48] Hu J , Chen Z , Xia D et al. Promoter-associated small double-stranded RNA interacts with heterogeneous nuclear ribonucleoprotein A2/B1 to induce transcriptional activation. Biochem J 2012; 447: 407–416. 23035981 10.1042/BJ20120256

[bib49] Torosyan Y , Dobi A , Glasman M et al. Role of multi-hnRNP nuclear complex in regulation of tumor suppressor ANXA7 in prostate cancer cells. Oncogene 2010; 29: 2457–2466. 20190808 10.1038/onc.2010.2

[bib50] Bannister AJ , Kouzarides T . The CBP co-activator is a histone acetyltransferase. Nature 1996; 384: 641–643. 8967953 10.1038/384641a0

[bib51] Honig B , Nicholls A . Classical electrostatics in biology and chemistry. Science 1995; 268: 1144–1149. 7761829 10.1126/science.7761829

