# Correction to: HnRNPA2 is a novel histone acetyltransferase that mediates mitochondrial stress-induced nuclear gene expression

**DOI:** 10.1038/s41421-019-0097-7

**Published:** 2019-05-14

**Authors:** Manti Guha, Satish Srinivasan, Kip Guja, Edison Mejia, Miguel Garcia-Diaz, F Brad Johnson, Gordon Ruthel, Brett A Kaufman, Eric F Rappaport, M Rebecca Glineburg, Ji-Kang Fang, Andres J Klein-Szanto, Hiroshi Nakagawa, Jeelan Basha, Tapas Kundu, Narayan G Avadhani

**Affiliations:** 1 Department of Biomedical Sciences & Mari Lowe Center for Comparative Oncology, School of Veterinary Medicine, University of Pennsylvania, Philadelphia, PA USA; 2 Department of Pharmacological Sciences, Stony Brook University, Stony Brook, NY USA; 3 Department of Pathology and Laboratory Medicine, Perelman School of Medicine, University of Pennsylvania, Philadelphia, PA USA; 4 Penn Vet Imaging Core, School of Veterinary Medicine, University of Pennsylvania, Philadelphia, PA USA; 5 Vascular Medicine Institute, University of Pittsburg, Pittsburgh, PA USA; 6 Nucleic Acid/Protein Core Facility, Children’s Hospital of Philadelphia Research Institute, Philadelphia, PA USA; 7 Histopathology Facility, Fox Chase Cancer Center, Temple University, Philadelphia, PA USA; 8 Department of Gastroenterology, Perelman School of Medicine, University of Pennsylvania, Philadelphia, PA USA; 9 Transcription and Disease Laboratory, Molecular Biology and Genetics Unit, Jawaharlal Nehru Centre for Advanced Scientific Research, Jakkur, Bangalore, India

**Correction to: Cell Discovery** (2016) 2

10.1038/celldisc.2016.45 Published online 6 December 2016

The original version of this article unfortunately contained a mistake. The given and family name of Andres Klein Szanto is incorrect. The correct name is Andres J Klein-Szanto.

The original article has been corrected.

