# Supplementary material for: HnRNPA2 is a novel histone acetyltransferase that mediates mitochondrial stress-induced nuclear gene expression
Source: Cell Discov. 2016 Dec 6;2:16045–. doi: 10.1038/celldisc.2016.45 (PMC5148442; doi:10.1038/celldisc.2016.45)
Supplement: Supplementary Information [file celldisc201645-s1.pdf]

SUPPLEMENTARY INFORMATION

RT[2] Profiler^{TM} PCR Array

Total RNA was extracted from the C2C12 cell lines as indicated in the figure. The RT[2] Profiler, pre-designed PCR array for the cancer pathways was used to simultaneously examine the mRNA levels of 84 genes closely associated with oncogenesis, including “housekeeping genes” in 96-well plates following the manufacturer’s protocol (catalog number PAMM-033Z, SA Biosciences, Qiagen, CA). For analysis, the manufacturer supplied web-based analysis program was used. For analysis, the Hsp90 expression remained unchanged in all cell lines and was used as the endogenous control. The resulting values were reported as fold change. The negative controls ensured a lack of DNA contamination and set the threshold for the absent/present calls.

FIGURE LEGENDS

Figure S1. An alternate method of mtDNA depletion to activate retrograde signaling (A) Real time PCR showing relative mRNA levels of *Tfam* in C2C12 cells expressing shRNA against either *GFP* (negative control) or *Tfam*. (*Inset*) Western immunoblot showing *Tfam* protein levels in these cells. **(B)** (Left panel) Relative MtDNA content assessed from total DNA by real time PCR using primers for MtDNA coded gene (COXI) or nuclear coded single copy gene (CcOIVi1) in C2C12 cells expressing *Tfam* shRNA (left panel) and compared to the negative control cells expressing *GFP*shRNA. (Right panel) Relative mtDNA content in control, EtBr treated and reverted C2C12 cells. **(C)** Real time PCR analysis showing relative mRNA levels (compared to control cells) of retrograde signaling marker genes in *Tfam* shRNA expressing C2C12cells. *β Actin* was used as endogenous control for normalization. **(D)** Real time PCR showing relative mRNA levels of *Tfam* in MCF10A cells (*Left panel*). Relative MtDNA

content in MCF10A cells expressing shRNA against either *GFP or Tfam* (*Left panel*). **(E)** Transcript levels of *Tfam* (*Left panel*) and relative mtDNA content (*Right Panel*) in control and Akt knock out MEF cells and expressing shRNA against either *Gfp* or *Tfam* in these cells**.** Data are represented as mean ± SD.

Figure S2. KAT activity of hnRNPA2. (A and D)H4 acetylation by *in vitro* translated product (Tr.P) **(A)** or recombinant purified hnRNPA2**(D)** was detected using a commercially available (Active Motif, cat # 56100) fluorescence KAT assay kit that detects CoA-SH. **(B)** HnRNPA2 KAT activity detected by ELISA using biotinylated H4, acetyl CoA and anti-acetyl lysine antibody**. (C)** Purification profile of 6xHis- hnRNPA2. Coomassie staining pattern of the recombinant hnRNPA2 constructs purified through a HisTrap FF affinity column and run on a 12% SDS-PAGE **(E)** Autoacetylation of hnRNPA2 by filter binding assay using 6x His-hnRNPA2 and 3H acetyl-CoA**. (F)** Acetylation reactions were carried out using different concentrations of ^{14}C acetyl-CoA and 6xHis-hnRNPA2 as indicated. The reaction products were run on 14% SDS-PAGE and immunoblotted using anti-acetyl-lysine and anti-His-tag antibodies. **(G)** Filter binding KAT assays were carried out in a 30 µl reaction mixture (as described in Experimental procedures) with 100nM hnRNPA2 and hnRNPA1 as indicated. Purified hnRNPA1 was obtained in a buffer containing 6M urea. For comparison of KAT activities between A2 and A1 equimolar urea was added to hnRNPA2 in parallel reactions. Purified freeze-thawed A2 protein was used in a parallel KAT reaction as a negative control to confirm the enzymatic nature of the acetylation reaction. **(H)** Western Immunoblot showing that hnRNPA1 and A2 in control, MtDNA-depleted and MtDNA-depl/hnRNPA2sh C2C12 cells. **(I)** Inhibition of H4 acetylation by hnRNPA2 wild type and phospho mutants (T98A and S219A) using ^{14}C acetyl-CoA and H4

(1-19) peptide as substrates. (**J**) Inhibition of H4 acetylation by hnRNPA2 (full-length and RNA binding domain) using 14C acetyl-CoA in the presence of 50 μM synthetic KAT inhibitors (as indicated) Data are represented as mean ± SD.

Figure S3. Functional Validation of hnRNPA2 HAT function. (A) ChIP assay in control, mtDNA-depleted and reverted C2C12 cells showing enrichment levels of hnRNPA2, H4acetylation H4K8 acetylation and H4K5acetylation at the Cathepsin L promoter. **(B)** ChIP assay in control, MtDNA-depleted and reverted cells showing hnRNPA2 enrichment on RyR1 and Akt1 promoters as indicated. **(C)** *Left Panel*: ChIP assay showing H4K8 acetylation levels at the β Actin Promoter in control, MtDNA-depleted (EtBr treated and *Tfam* sh), reverted and MtDNA-depl/hnRNPA2sh cells. *Middle Panel*: ChIP assay of intronic regions of Cathepsin L and Akt1 genes showing H4K8 acetylation levels in control, MtDNA-depleted C2C12 cells. *Right Panel*: Table showing fold enrichment of H4K8 acetylation in target gene promoters and introns in mtDNA depleted cells over control. **(D)** Involvement of the hnRNPA2 KAT motifs in H4 acetylation at *Cathepsin L* promoter was detected by ChIP assay in MtDNA-depleted/hnRNPA2sh cells ectopically expressing either the pMXs vector alone or hnRNPA2 WT, R48T, R50T, and R48T/R50T mutants. **(E)** Promoter activity of pGL3-Cathepsin L promoter in MtDNA-depleted/hnRNPA2sh cells ectopically expressing hnRNPA2 WT, R48T, R50T, and R48T/R50T mutants. The pGL3 vector alone was used as negative control. *Renilla* luciferase activity was used as an internal control for transfection efficiency. Data are represented as mean ± SD.

Figure S4. Altered Gene Expression pattern in hnRNPA2 KAT mutant cells. Heat Map Clustergram showing global alterations in gene expression patterns between control, MtDNA-depleted, MtDNA-depl/hnRNPA2sh cells and MtDNA-depl/hnRNPA2sh cells expressing either wild type or KAT mutant hnRNPA2.

Legend for Supplementary Table S1:

List of genes (expressed as Fold Change with respect to control) altered in PMtDNA-depleted, PMtDNA-depl/hnRNPA2sh C2C12 cells ectopically expressing hnRNPA2 WT and KAT mutants. Among the 9 endogenous control genes on the RT2 Profiler Array, HSP90 was unchanged for all our samples and was selected for normalization of the Real Time PCR values for all samples.

PRIMER SEQUENCES

Promoter DNA primers for ChIP Assay:
mAkt1Pro-F: ACACTCCTGGTGAACTCTGACCCT
mAkt1Pro-R: GGTGGCCCCGAGCCAGGGCCACGC

mRyR1Pro-F: AGTTCACTGGAGAGAGAA
mRyR1Pro-R: TGGAACTGCAGGAAGCAT
mCathepsin LPro-F: GAGGTGGAAATTCCAC
mCathepsin LPro-R: CCCGCCCCCGCCCCG

Cathepsin L-INTRONCHIP-F: CAGCACCCTCTGCTTTTCAGT
Cathepsin L-INTRONCHIP-R: TGGCTCAACAGGGAAAGGAA

AKT1-INTRONCHIP-F: TTCAGGGCTATTTGGGCAAA
AKT1-INTRONCHIP-R: AGCACAAACAGAGCTCCTAGGAA

Internal control primers for telomere PCR:
36B4 Forward Primer: ACT GGT CTA GGA CCC GAG AAG
36B4 Reverse Primer: TCA ATG GTG CCT CTG GAG ATT
Telomere Repeat Amplification Protocol (QTRAP) Assay

TS primer: 5'-AATCCGTCGAGCAGAGTT
ACX primer: 5'-GCGCGGCTTACCCTTACCCTTACCCTAACC

REFERENCES

Guha,M., Pan,H., Fang,J.K., and Avadhani,N.G. (2009). Heterogeneous nuclear ribonucleoprotein A2 is a common transcriptional coactivator in the nuclear transcription response to mitochondrial respiratory stress. Mol. Biol. Cell *20*, 4107-4119.

Guha,M., Srinivasan,S., Ruthel,G., Kashina,A.K., Carstens,R.P., Mendoza,A., Khanna,C., Van,W.T., and Avadhani,N.G. (2013). Mitochondrial retrograde signaling induces epithelial-mesenchymal transition and generates breast cancer stem cells. Oncogene, 33(45):5238-50.

Liu,X., Wang,L., Zhao,K., Thompson,P.R., Hwang,Y., Marmorstein,R., and Cole,P.A. (2008). The structural basis of protein acetylation by the p300/CBP transcriptional coactivator. Nature *451*, 846-850.

Srinivasan, S., Guha M, Dong DW, Whelan K, Ruthel G, Uchikado Y, Natsugoe S, Nakagawa, H., and Avadhani, N. G. Disruption of Cytochrome c Oxidase Function Induces Warburg Effect and Metabolic Reprogramming. Oncogene . 2015. Jul 6. doi: 10.1038/onc.2015.227. [Epub ahead of print]

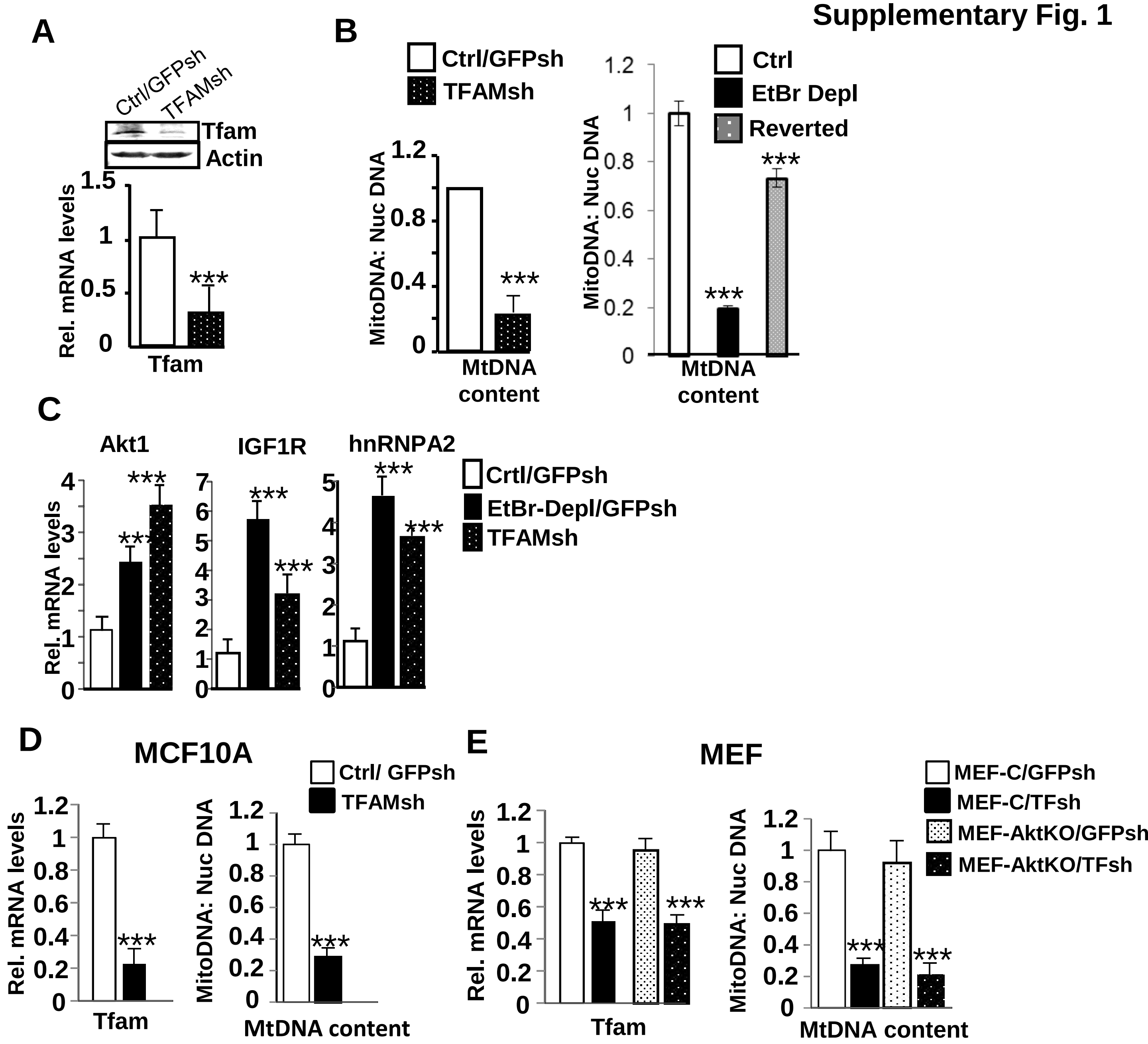

Figure S1. An alternate method of mtDNA depletion to activate retrograde signaling (A) Real time PCR showing relative mRNA levels of Tfam in C2C12 cells expressing shRNA against either GFP (negative control) or Tfam. (Inset) Western immunoblot showing Tfam protein levels in these cells. (B) (Left panel) Relative MtDNA content assessed from total DNA by real time PCR using primers for MtDNA coded gene (COXI) or nuclear coded single copy gene (CcOIVi1) in C2C12 cells expressing Tfam shRNA (left panel) and compared to the negative control cells expressing GFPshRNA. (Right panel) Relative mtDNA content in control, EtBr treated and reverted C2C12 cells. (C) Real time PCR analysis showing relative mRNA levels (compared to control cells) of retrograde signaling marker genes in Tfam shRNA expressing C2C12cells. β Actin was used as endogenous control for normalization. (D) Real time PCR showing relative mRNA levels of Tfam in MCF10A cells (Left panel). Relative MtDNA content in MCF10A cells expressing shRNA against either GFP or Tfam (Left panel). (E) Transcript levels of Tfam (Left panel) and relative mtDNA content (Right Panel) in control and Akt knock out MEF cells and expressing shRNA against either Gfp or Tfam in these cells. Data are represented as mean ± SD.

Supplementary Fig. 2

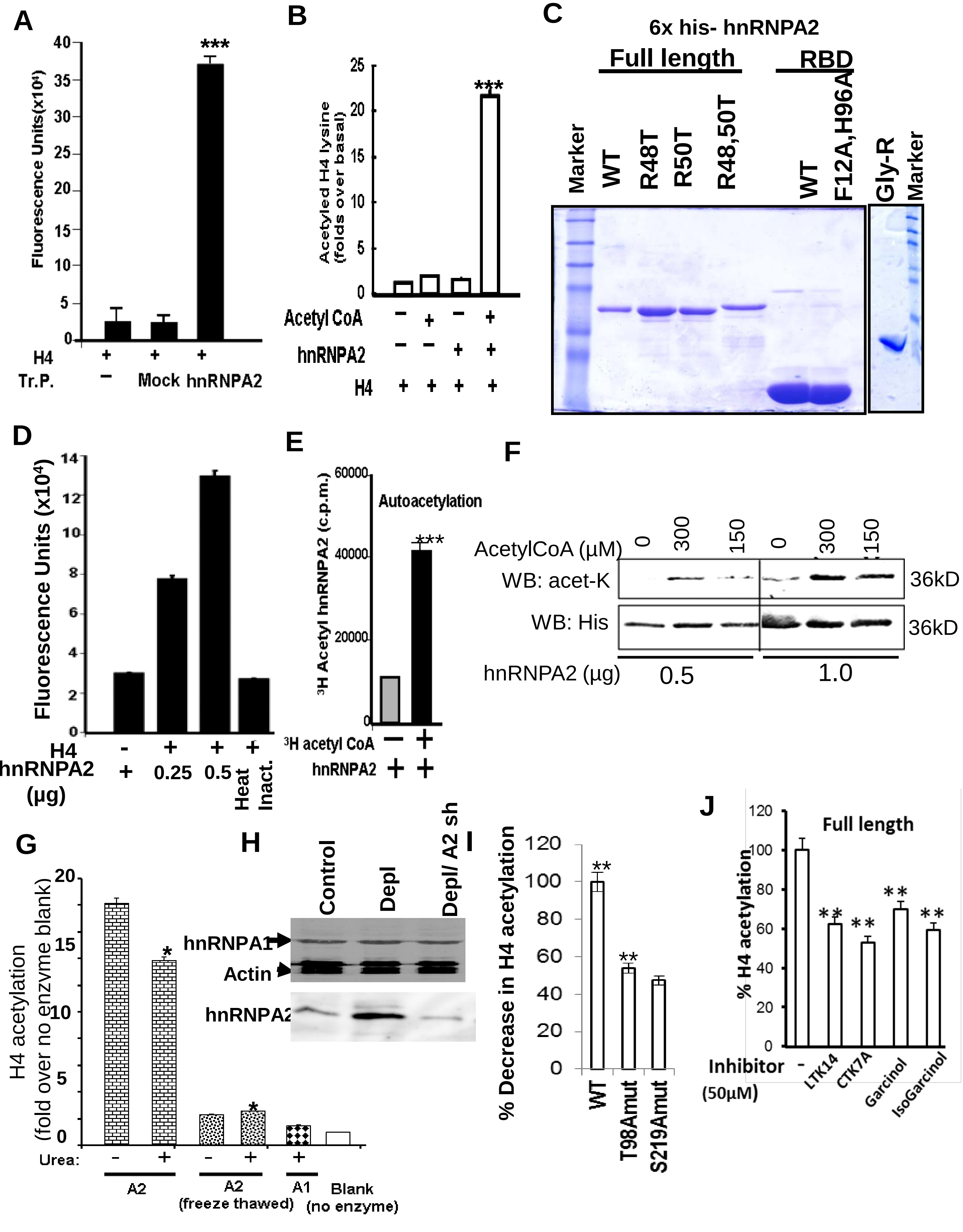

Supplementary Fig. 3

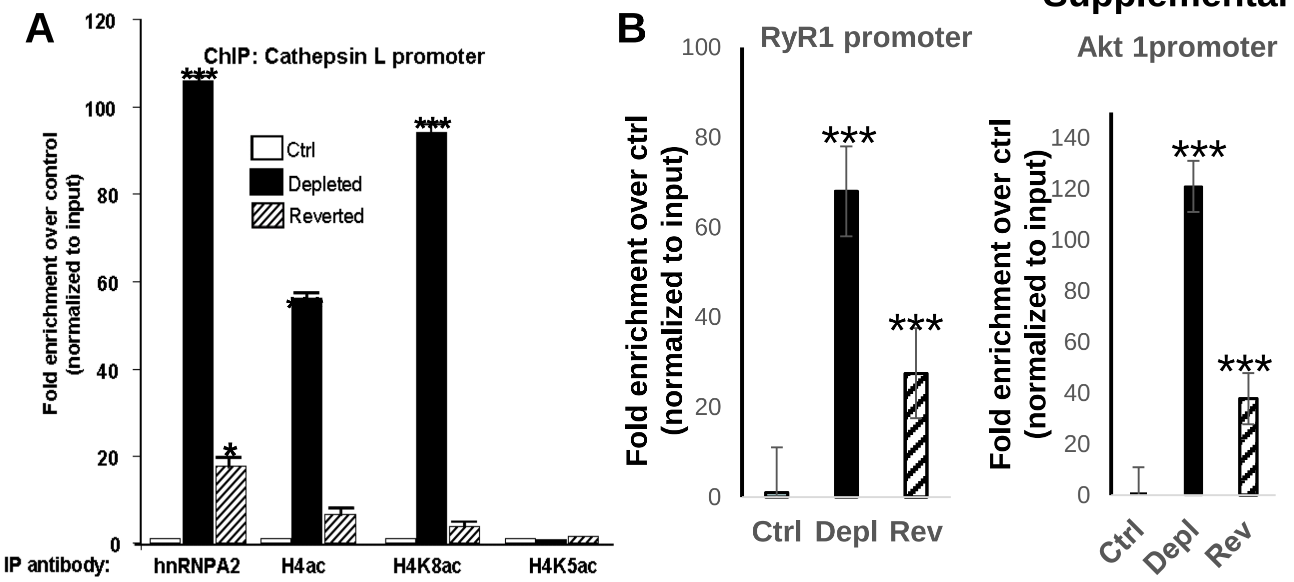

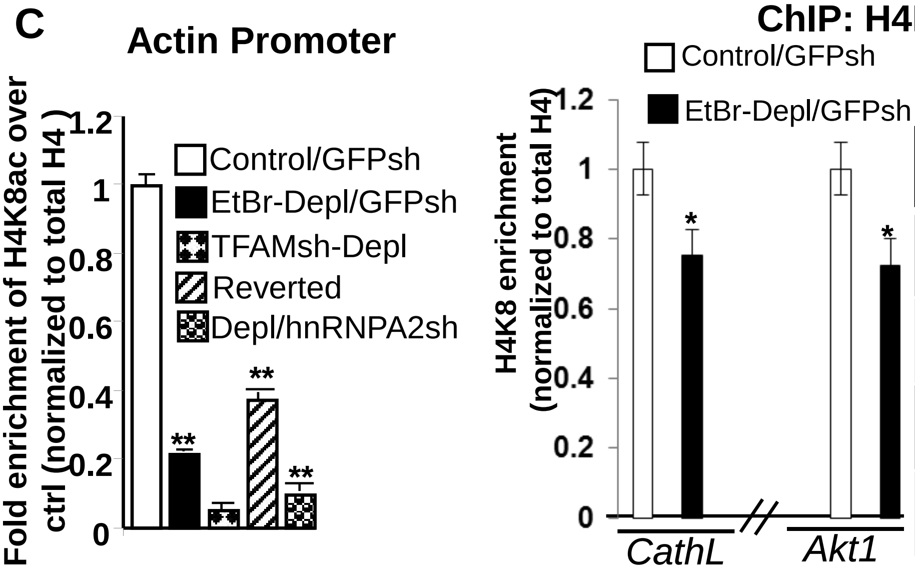

Fold Enrichment of H4K8ac

	Ctrl	EtBR-Depl
Akt1-Promoter	1	47.15
Akt1-Intron	1	0.75187
Cath L - Promoter	1	90.247
Cath L-Intron	1	0.723985

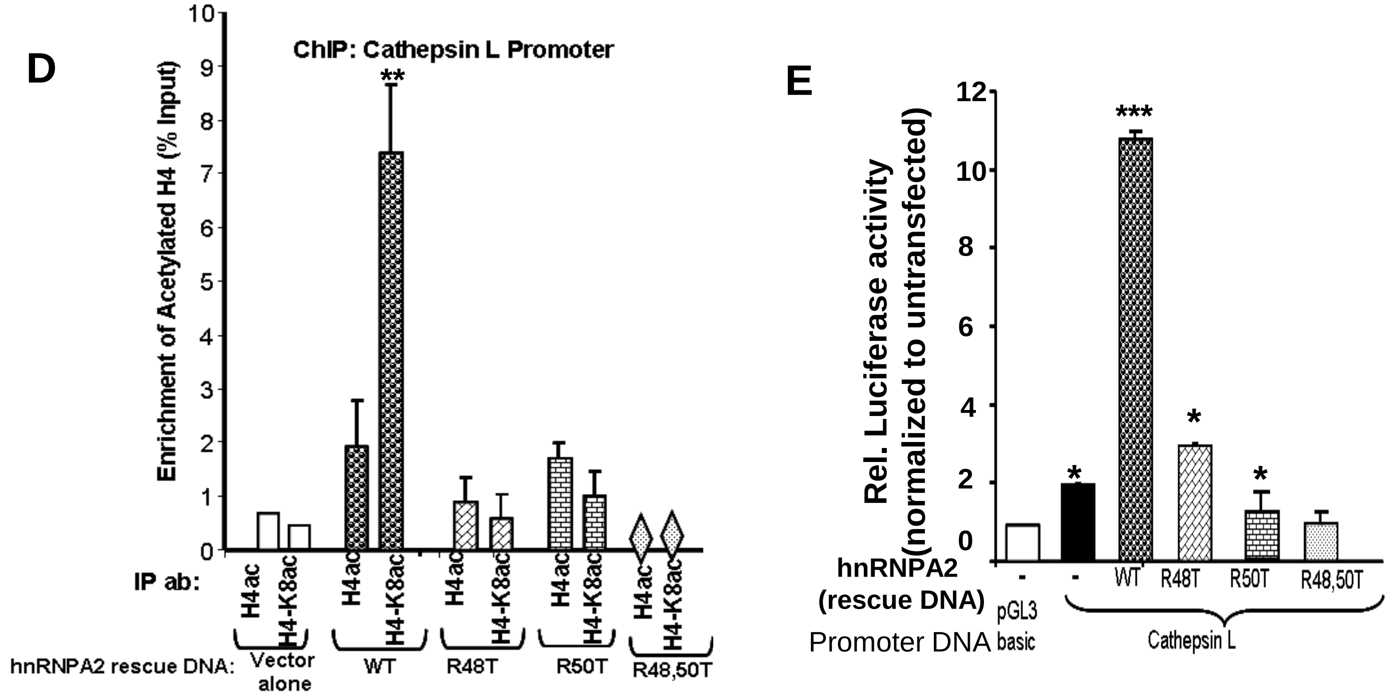

Supplementary Fig. 4

Clustergram

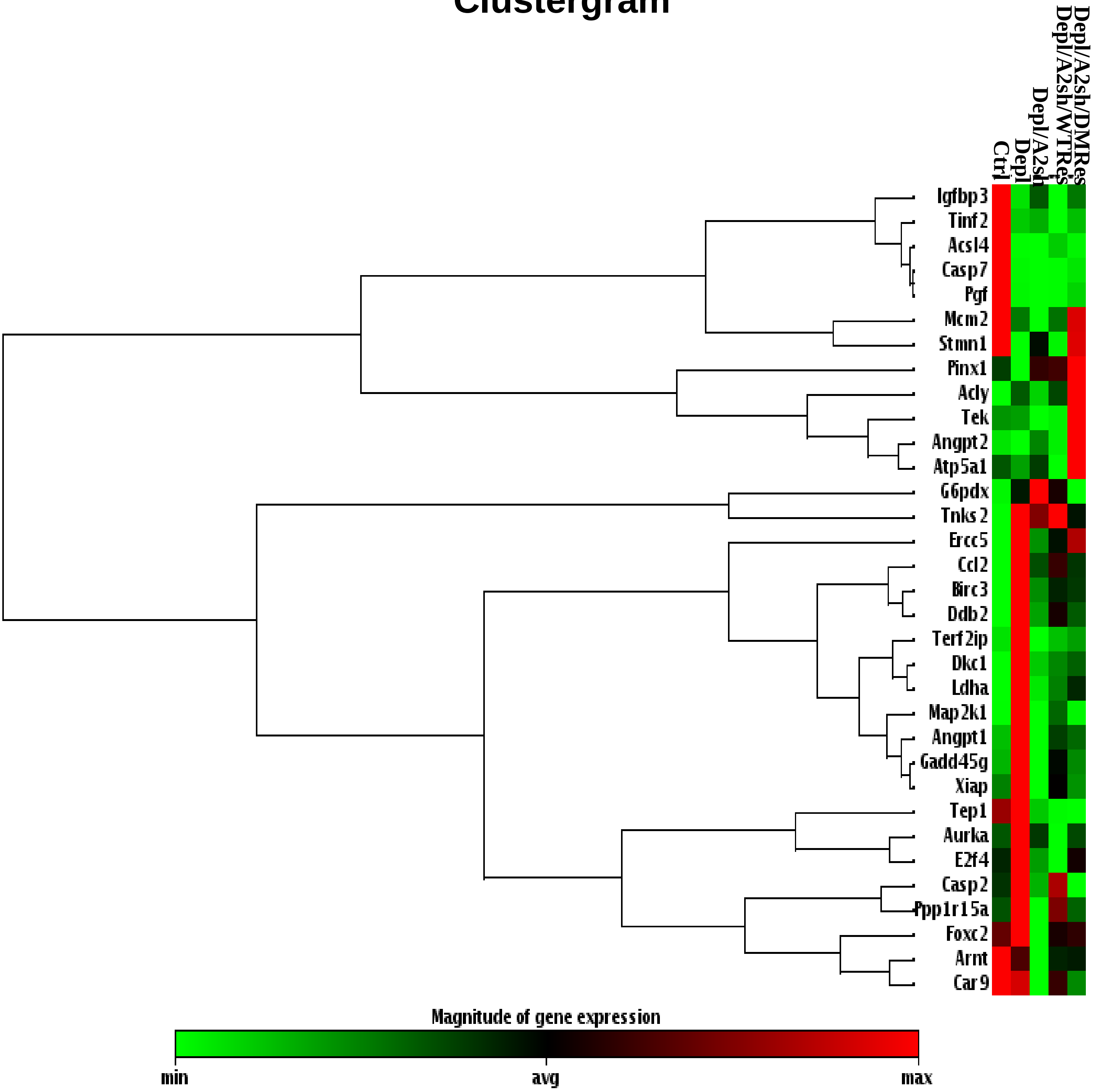

Figure S4. Altered Gene Expression pattern in hnRNPA2 KAT mutant cells. Heat Map Clustergram showing global alterations in gene expression patterns between control, MtDNA-depleted, MtDNA-depl/hnRNPA2sh cells and MtDNA-depl/hnRNPA2sh cells expressing either wild type or KAT mutant hnRNPA2.
