# Supplementary material for: HnRNPA2 is a novel histone acetyltransferase that mediates mitochondrial stress-induced nuclear gene expression
Source: Cell Discov. 2016 Dec 6;2:16045–. doi: 10.1038/celldisc.2016.45 (PMC5148442; doi:10.1038/celldisc.2016.45)
Supplement: Supplementary Table S1 [file celldisc201645-s2.pdf]

Table S1: Fold changes in gene expression over control cells.

Gene Symbol	Depl	Depl/A2sh	Depl/A2sh /WT Res	Depl/A2sh /HATmutRes
Acly	1.454	1.1173	1.5052	0.9931
Acsl4	0.1406	0.1387	0.2207	0.0954
Angpt1	4.2281	0.5471	1.9453	0.6329
Angpt2	0.9593	1.1567	0.9794	1.3287
Birc3	10.3388	3.0738	5.063	2.6027
Casp2	1.5801	0.7526	1.4241	0.6113
Ccl2	12.4666	4.9588	7.9447	4.1699
Ddb2	2.3457	1.2397	1.7291	1.434
Dkc1	8.8152	1.7901	2.8284	1.4142
Ercc5	3.4822	1.5263	2.1585	1.6702
Foxc2	1.257	0.4061	0.8706	0.5249
Gadd45g	3.1821	0.6373	1.8661	0.5987
Igfbp3	0.1528	0.3978	0.1051	0.3415
Map2k1	4.5631	0.9931	2.0705	1.0425
Ppp1r15a	2.0994	0.4383	1.6702	0.5249
Stmn1	0.3345	0.6507	0.3463	0.9593
Terf2ip	2.639	0.9075	1.1173	0.6071
Tinf2	0.3511	0.3923	0.2793	0.4263
Tnks	0.8586	0.4005	0.732	0.2606
Tnks2	1.4241	1.3195	1.4241	0.9138
Xiap	1.4142	0.8645	1.1408	0.7684
